# Synthesis and biological evaluation of 2-aryl-benzimidazole derivatives of dehydroabietic acid as novel tubulin polymerization inhibitors†

**DOI:** 10.1039/c8ra02078g

**Published:** 2018-05-16

**Authors:** Ting-Ting Miao, Xu-Bing Tao, Dong-Dong Li, Hao Chen, Xiao-Yan Jin, Yi Geng, Shi-Fa Wang, Wen Gu

**Affiliations:** Jiangsu Provincial Key Lab for the Chemistry and Utilization of Agro-forest Biomass, Jiangsu Key Lab of Biomass-based Green Fuels and Chemicals, College of Chemical Engineering, Nanjing Forestry University Nanjing 210037 P. R. China njguwen@163.com

## Abstract

A series of novel 2-aryl-benzimidazole derivatives of dehydroabietic acid were synthesized and characterized by IR, ^1^H NMR, ^13^C NMR, MS and elemental analyses. All the target compounds were evaluated for their *in vitro* cytotoxic activity against SMMC-7721, MDA-MB-231, HeLa and CT-26 cancer cell lines and the normal hepatocyte cell line QSG-7701 through MTT assays. Among them, compound 6j displayed the most potent cytotoxic activity with IC_50_ values of 0.08 ± 0.01, 0.19 ± 0.04, 0.23 ± 0.05 and 0.42 ± 0.07 μM, respectively, and substantially reduced cytotoxicity against QSG-7701 cells (5.82 ± 0.38 μM). The treatment of SMMC-7721 cells with compound 6j led to considerable inhibition of cell migration ability. The influence of compound 6j on cell cycle distribution was assessed on SMMC-7721 cells, exhibiting a cell cycle arrest at the G2/M phase. Moreover, tubulin polymerization assays and immunofluorescence assays elucidated that compound 6j could significantly inhibit tubulin polymerization and disrupt the intracellular microtubule network. A molecular docking study provided insight into the binding mode of compound 6j in the colchicine site of tubulin. In addition, compound 6j was found to induce apoptosis of SMMC-7721 cells, an increase of intracellular ROS level and a loss of mitochondrial membrane potential in a dose-dependent manner. These findings provided new molecular scaffolds for the further development of novel antitumor agents targeting tubulin polymerization.

## Abstract 2

A series of novel 2-aryl-benzimidazole derivatives of dehydroabietic acid were synthesized. Among them, compound 6j was found to be a potent tubulin polymerization inhibitor.

## Graphical abstract

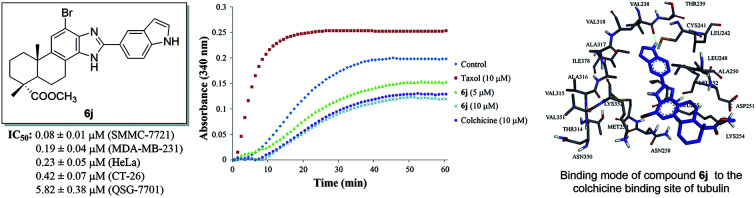

## Introduction

Microtubules, as key components of the cytoskeleton, are dynamic polymers of α, β-tubulin and involved in a series of important cellular processes including regulation of motility, cell signaling, maintenance of cell shape, cell proliferation and intracellular transport.^1–4^ Due to the multiple functions of microtubules in the cell cycle, tubulin has become an attractive target for the development of novel antimitotic agents for cancer therapy.^5,6^ Antimitotic agents are generally divided into two types: microtubule stabilizing agents (*e.g.*, paclitaxel, epothilones) and tubulin polymerization inhibitors (*e.g.*, vinca alkaloids, colchicine). Three major binding sites on microtubules have been identified as the taxane-, vinca alkaloid- and colchicine-binding sites.^7^ Disruption of microtubule dynamics by these compounds will lead to cell cycle arrest at G2/M phase and induction of apoptosis.^8^

**Fig. 1:**
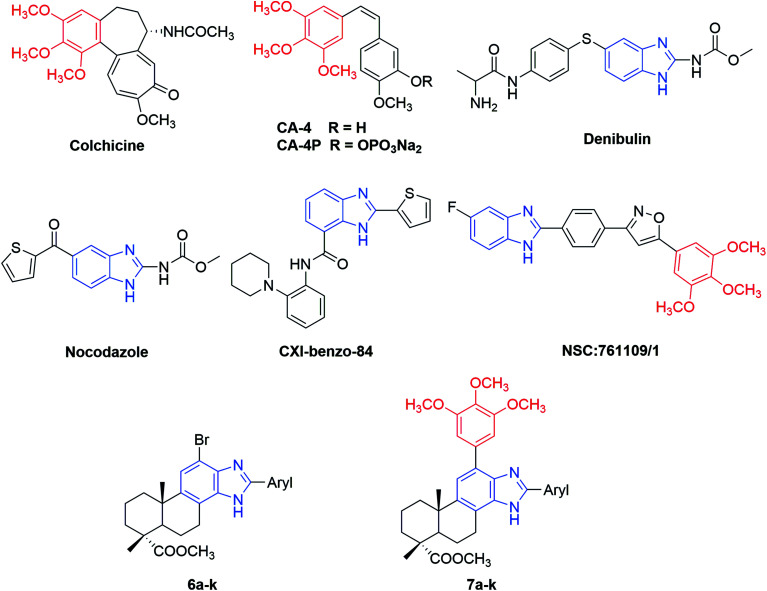
Structures of some reported antimitotic agents targeting colchicine-binding site and the newly synthesized 2-aryl-benzimidazole derivatives of dehydroabietic acid (6a–k and 7a–k).

In the past several decades, antimitotic agents interacting with the taxane- or vinca alkaloid-binding sites have acquired tremendous success in clinical oncology. However, compounds targeting the colchicine-binding site have drawn increasing interest from medicinal chemists in recent years for their high anticancer potency, selective toxicity toward tumor vasculature and promising ability to overcome P-glycoprotein (P-gp) efflux pump mediated multidrug resistance.^9–11^ For example, combretastatin A-4 (CA-4) (Fig. 1) is a natural (*Z*)-stilbene isolated from the South African bush willow tree *Combretum caffrum* by Pettit and co-workers in 1989.^12^ Through binding to the colchicine site, CA-4 can significantly inhibit tubulin polymerization and exhibit a potent cytotoxicity at nanomolar level against a wide range of human cancer cells. Moreover, CA-4 displayed a selective toxicity towards the vascular network of tumors, inducing an irreversible shutdown of blood flow to neoplastic cells.^13^ Some water soluble prodrugs of CA-4 were subjected to clinical trials,^14^ among which the phosphate disodium prodrug (CA-4P, fosbretabulin, Zybrestat™) received the orphan drug status (USA and Europe) in 2016 to treat neuroendocrine tumors (NETs) and glioblastoma multiform (GBM).^15,16^ Therefore, antimitotic agents targeting the colchicine-binding site have proved to be a promising source for the development of novel and potent anticancer drugs.

Nitrogen-containing heterocycles play a vital role in the field of medicinal chemistry because they can easily interact with biomolecules of living systems.^17^ Among them, benzimidazole has been considered as a prominent heterocyclic scaffold found in many natural and synthetic drugs which exhibit a wide range of therapeutic properties.^18^ As antitumor agents, benzimidazole derivatives can exert their antitumor activity by acting on various targets such as topoisomerase inhibitors, DNA intercalating agents, androgen receptor antagonists, antiangiogenic agents, poly(ADP-ribose) polymerase (PARP) inhibitors, dihydrofolate reductase inhibitors, protein tyrosine kinase inhibitors and tubulin polymerization inhibitors.^19–25^ For example, nocodazole (NSC-238189), with benzimidazole as its basic scaffold, is a well-known anticancer agent that significantly inhibits the polymerization of tubulin at nanomolar concentration.^26^ Besides nocodazole, a number of benzimidazole derivatives such as denibulin,^27^ CXI-benzo-84,^28^ NSC:761109/1,^29^*etc.* have also been reported as potent tubulin polymerization inhibitors binding to the colchicine site (Fig. 1). On the other hand, some above-mentioned compounds such as colchicine and CA-4 shared a common 3,4,5-trimethoxyphenyl (TMP) moiety in their chemical structures, implying this moiety might be crucial for their anti-tubulin polymerization activities.^30^ These findings suggested that benzimidazole and TMP moieties could be useful pharmacophores for the design of novel tubulin polymerization inhibitors.

Natural products provide a rich source for new medicinal leads and play a major role in drug discovery, especially in the area of cancer therapy.^31^ Dehydroabietic acid (DAA, 1) is a natural diterpenic resin acid which can be easily isolated from *Pinus* rosin or commercial disproportionated rosin. DAA and its derivatives has been found to exhibit a broad spectrum of biological activities, such as antimicrobial, antiprotozoal, antiviral, antiulcer, anti-aging and radical scavenging activities.^32–37^ Especially, a lot of DAA derivatives have been reported exhibiting prominent anticancer activity through various mechanisms including DNA binding,^38^ induction of apoptosis^39^ or oncosis^40^ and inhibition of cancer cell migration.^41^ These results suggest that dehydroabietic acid is a promising lead compound for the investigation of new anticancer agents, and the incorporation of benzimidazole and TMP moieties to the structure of DAA may result in novel hybrid molecules with improved antimitotic properties. In continuation of our research on novel bioactive derivatives of DAA,^42,43^ a series of new 2-aryl-benzimidazole derivatives of DAA were designed and synthesized, which were assayed for their *in vitro* antiproliferative activity against several cell lines. In addition, the possible anticancer mechanisms of these derivatives as tubulin polymerization inhibitor and apoptosis inducer were also preliminarily explored.

## Results and discussion

### Chemistry

**Scheme 1:**
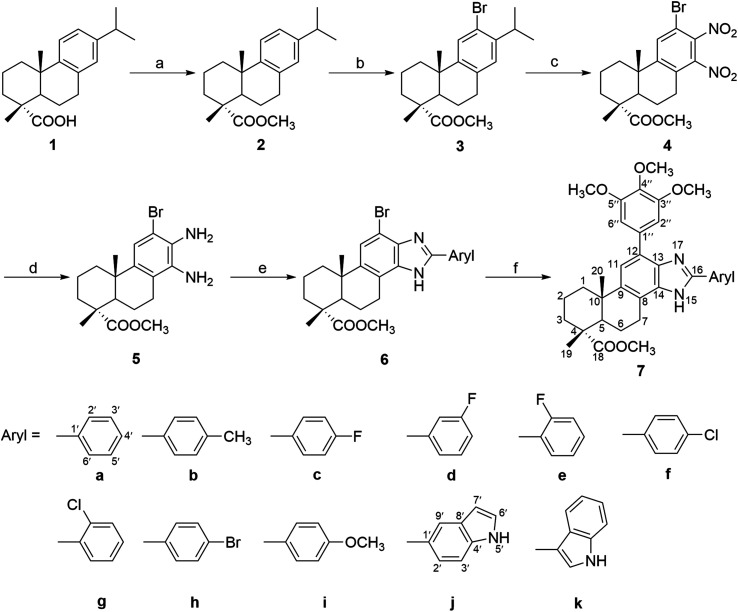
Reagents and conditions: (a) (i) SOCl_2_, benzene, reflux, 3 h, (ii) MeOH, reflux, 5 h; (b) NBS, MeCN, rt, 24 h; (c) fuming HNO_3_, H_2_SO_4_, 0 °C, 40 min; (d) Fe, EtOH, H_2_O, HCl, reflux, 4 h; (e) substituted arylaldehyde, *p*-toluenesulfonic acid, EtOH, 85 °C, 24 h; (f) 3,4,5-trimethoxy-phenylboronic acid, Pd(PPh_3_)_4_, K_2_CO_3_, THF, H_2_O, 85 °C, 24 h.

The target compounds (6a–k and 7a–k) were synthesized according to the method outlined in Scheme 1. The key intermediate methyl 12-bromo-13,14-dinitro-deisopropyldehydroabietate (4) was synthesized from dehydroabietic acid based on the procedure reported in a previous literature.^44^ Subsequently, two nitro groups of compound 4 were reduced by Fe powder to afford the diamino derivatives 5 in 57% yield, which was then reacted with different substituted arylaldehyde to get a series of 2′-aryl-12-bromo-benzimidazole derivatives (6a–k) in 60–83% yield. Furthermore, compounds 6a–k were reacted with 3,4,5-trimethoxy-phenylboronic acid in the presence of Pd(PPh_3_)_4_ and K_2_CO_3_ to afford the corresponding 2′-aryl-12-(3,4,5-trimethoxyphenyl)-benzimidazole derivatives (7a–k) in 50–73% yield. All the synthesized compounds were purified by recrystallization or silica gel column chromatography.

The structures of compounds 6a–k and 7a–k were characterized on the basis of MS, IR, ^1^H NMR, ^13^C NMR and elemental analyses. In a typical example, the ESIMS of compound 6j displayed two peaks at *m*/*z* 506 and 508, corresponding to the quasimolcular ions [M + H]^+^, whcih also suggested the presence of a bromine atom. The data of molecular weight in combination with the elemental analysis confirmed the molecular formula of compound 6j as C_27_H_28_BrN_3_O_2_. The IR spectrum of 6j exhibited a broad strong peaks at 3325 cm^−1^ corresponding to the N–H stretch vibrations and two strong C–H vibration bands at 2929 and 2866 cm^−1^ attributed to the C–H stretch vibrations. In addition, a very strong absorption band at 1703 cm^−1^ was due to the C=O stretch vibrations of the methyl ester moiety. The ^1^H-NMR spectrum of 6j showed three singlets at *δ* 1.28, 1.32 and 3.69 ppm, attributed to methyl protons at C-19, C-20 and 18-ester group, respectively. The peaks in the range of *δ* 1.50–3.20 ppm could be attributed to the eleven protons of five aliphatic methylenes and an aliphatic methine at C-5. The six peaks appeared at *δ* 6.62, 7.28, 7.32, 7.45, 7.90 and 8.29 ppm could be assigned as the aromatic protons in the benzimidazole and indole moieties, and the N–H signals appeared as a broad single peak at *δ* 8.44 ppm. In the ^13^C-NMR spectrum of 6j, there were 27 peaks appearing in the range from *δ* 16–180 ppm. Among them, fifteen peaks at *δ* 100–155 ppm, including six tertiary carbons and nine quaternary carbons, were due to the aromatic carbons on the benzimidazole and indole moieties. In addition, The peaks at *δ* 52.2 and 179.5 ppm could be attributed to the signals of methyl carbon and carbonyl carbon on C-18 ester group, respectively. The assignments of the signals in the ^1^H and ^13^C NMR spectra of compound 6j were in good accordance with its structure. As for compound 7j, its ESIMS spectrum and elemental analysis data also confirmed its molecular formula C_36_H_39_N_2_O_5_. The ^1^H NMR spectrum of 7j exhibited two singlets at *δ* 3.93 (OCH_3_) and 3.96 ppm (OCH_3_ × 2) and a 2H singlet at *δ* 6.82 ppm (H-2′′ and H-6′′). The ^13^C NMR spectra of 7j also demonstrated six additional peaks compared with compound 6j. Among them, the peaks at *δ* 56.5 and 61.1 ppm were attributed to the three methoxyl groups in the benzene ring. These signals clearly indicated the presence of 3,4,5-trimethoxyphenyl moiety in compound 7j.

### Biological evaluation

#### *In vitro* antiproliferative activity

The *in vitro* antiproliferative activity of DAA derivatives (1–5, 6a–k, 7a–k) were evaluated by MTT assay against human hepatocarcinoma cell line (SMMC-7721), human breast cancer cell line (MDA-MB-231), human cervical carcinoma cell line (HeLa), mouse colon cancer cell line (CT-26) and human normal hepatocyte cell line (QSG-7701). Nocodazole was co-assayed as the positive control and DMEM culture medium containing 0.1% DMSO was used as negative control. The results of the tested compounds expressed as IC_50_ values (concentration required to inhibit tumor cell proliferation by 50%) were presented in Table 1.

**Table 1:** The *in vitro* cytotoxic activities of the tested compounds (1–5, 6a–k and 7a–k) against four cancer cell lines (SMMC-7721, MDA-MB-231, HeLa and CT-26) and one normal hepatocyte cell line (QSG-7701)

Compound	IC_50_ (μM)				
	SMMC-7721	MDA-MB-231	HeLa	CT-26	QSG-7701
1	>50	>50	>50	>50	NT^a^
2	>50	>50	>50	>50	NT
3	>50	>50	>50	>50	NT
4	31.80 ± 3.22	38.76 ± 2.15	>50	35.13 ± 3.87	NT
5	>50	>50	43.43 ± 2.81	38.52 ± 3.79	NT
6a	12.47 ± 0.45	41.72 ± 2.06	16.21 ± 1.73	6.34 ± 0.83	>50
6b	>50	>50	>50	25.26 ± 4.96	NT
6c	1.52 ± 0.77	18.21 ± 1.20	8.01 ± 0.76	2.82 ± 0.97	>50
6d	5.27 ± 0.27	11.97 ± 1.31	17.73 ± 3.02	3.39 ± 0.33	>50
6e	12.28 ± 2.46	>50	>50	>50	NT
6f	16.49 ± 1.87	33.66 ± 4.70	>50	>50	NT
6g	40.81 ± 3.32	19.21 ± 0.93	>50	>50	NT
6h	16.58 ± 1.40	35.26 ± 1.77	>50	>50	NT
6i	>50	>50	>50	21.23 ± 2.23	NT
6j	0.08 ± 0.01	0.19 ± 0.04	0.23 ± 0.05	0.42 ± 0.07	5.82 ± 0.38
6k	1.47 ± 0.08	0.56 ± 0.13	0.98 ± 0.11	0.67 ± 0.03	8.43 ± 1.06
7a	9.38 ± 1.02	>50	13.12 ± 1.70	8.25 ± 0.50	>50
7b	>50	>50	>50	15.60 ± 1.57	NT
7c	3.29 ± 0.13	0.69 ± 0.21	7.63 ± 1.03	8.95 ± 0.17	36.07 ± 2.32
7d	>50	6.42 ± 0.04	12.89 ± 1.26	2.70 ± 0.12	>50
7e	>50	18.20 ± 1.32	18.34 ± 2.98	10.41 ± 1.91	NT
7f	11.00 ± 2.68	10.14 ± 1.38	17.21 ± 2.32	3.44 ± 0.75	>50
7g	>50	15.74 ± 0.87	>50	13.43 ± 2.35	NT
7h	>50	8.84 ± 1.08	29.88 ± 3.07	2.76 ± 0.26	>50
7i	>50	>50	>50	23.52 ± 2.38	NT
7j	2.35 ± 1.09	0.83 ± 0.20	3.75 ± 0.78	0.59 ± 0.08	12.83 ± 1.27
7k	1.95 ± 0.56	0.21 ± 0.02	5.86 ± 0.63	0.45 ± 0.03	11.78 ± 2.12
Nocodazole	0.92 ± 0.07	0.76 ± 0.06	0.87 ± 0.05	1.37 ± 0.11	13.75 ± 1.08

a NT: Not tested.

From the results, the target compounds exhibited varying degrees of cytotoxic activity against four cancer cell lines. Specifically, compounds 6c, 6d, 6j, 6k, 7c, 7j and 7k showed significant anticancer activity against SMMC-7721 cells with IC_50_ values below 5 μM, compounds 6j, 6k, 7c, 7j and 7k displayed strong cytotoxicity against MDA-MB-231 cells, compounds 6j, 6k, 7j and 7k showed strong inhibition against HeLa cells, and compounds 6c, 6d, 6j, 6k, 7d, 7f, 7h, 7j and 7k exhibited pronounced activity against CT-26 cells (IC_50_ < 5 μM). In general, compounds 6a–k exhibited stronger activity against SMMC-7721 cells than the other three cancer cell lines, while compounds 7a–k were most cytotoxic to CT-26 cells. Among these derivatives, compound 6j exhibited the most potent anticancer activity against all the four cancer cell lines, with IC_50_ values of 0.08 ± 0.01, 0.19 ± 0.04, 0.23 ± 0.05 and 0.42 ± 0.07 μM, respectively, stronger than the positive control nocodazole. In addition, compounds 6k, 7j and 7k also exhibited potent cytotoxic activity at low μM range. It is worth noting that compounds 6j, 6k and 7j, 7k were significantly less cytotoxic to human normal hepatocyte cell line QSG-7701, which indicated that these compounds possessed potent and selective antiproliferative activity on the tested cancer cell lines.

Based on the above observation, some interesting structure–activity relationships (SAR) could be concluded. As shown in Table 1, the cytotoxic activities of most target derivatives (6a–k, 7a–k) with benzimidazole moieties were superior to DAA (1) and intermediates 2–5, which indicated that the introduction of benzimidazole moiety could significantly improve the cytotoxicity. However, compounds 7a–k did not exhibit much stronger cytotoxic activities than compounds 6a–k, suggesting that the displacement of 12-Br with 12-(3,4,5-trimethoxyphenyl) moiety were not obviously advantageous to anticancer activity as expected. On the other hand, the 2′-aryl groups on benzimidazole rings also significantly affected the cytotoxic potencies of the target compounds. For series 6a–k, compounds 6c, 6f, 6h with *p*-fluoro, *p*-chloro and *p*-bromo substituents on the benzene rings exhibited substantially stronger cytotoxic activity than compounds 6b and 6i with *p*-methyl and *p*-methoxy groups. In addition, compound 6c with *p*-fluoro group was more cytotoxic than 6d with *m*-fluoro group which was also more cytotoxic than 6e with *o*-fluoro group. The similar results could also be observed in series 7a–k. These results indicated that the electron-withdrawing groups (–F, −Cl and −Br) on the benzene rings will be more beneficial to the cytotoxic activity than electron-donating ones (−Me and −OMe), and the fluoro or chloro groups on *p*-position will also be more favorable to the cytotoxicity than those on *m*- or *o*-positions. Notably, compounds 6j, 6k and 7j, 7k exhibited much more potent anticancer activities than other derivatives, indicating that the presence of 1*H*-indol-5-yl and 1*H*-indol-3-yl moieties, especially 1*H*-indol-5-yl moiety, would be more propitious to the interaction between these derivatives and their potential target.

#### Cell cycle analysis

**Fig. 2:**
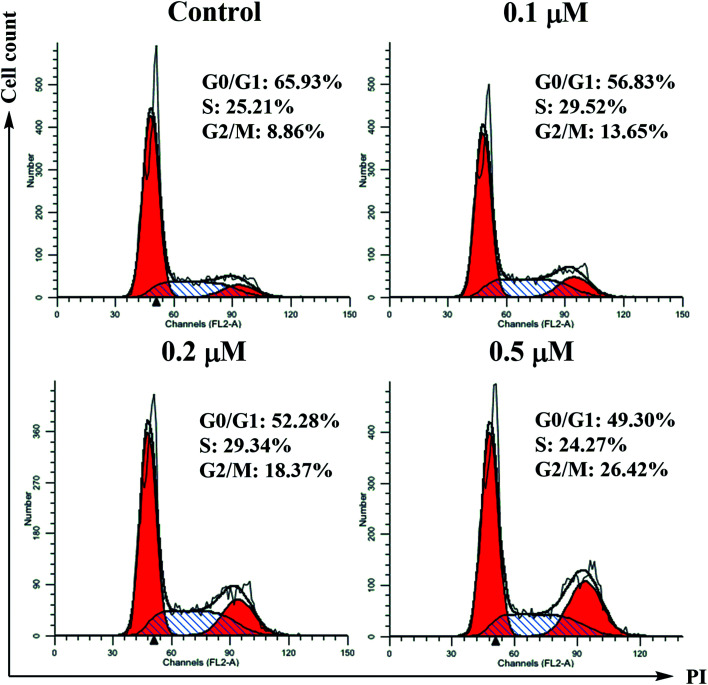
Effects of compound 6j on cell cycle progression of SMMC-7721 cells. The analysis of cell cycle distribution was performed by using propidium iodide staining method. Cells were treated with 0.1, 0.2 and 0.5 μM of 6j for 24 h.

Based on the results of the cytotoxic evaluation, compound 6j with the most potent cytotoxicity was chosen for the in-depth mechanism study. Flow cytometry was used to analyze the effects of the DAA derivative on cell growth and division. SMMC-7721 cells were treated with different concentrations of compound 6j (0, 0.1, 0.2 and 0.5 μM) for 24 h. Cell cycle distribution was investigated by flow cytometric analysis after staining the DNA of the treated cells with propidium iodide (PI). As shown in Fig. 2, the percentage of cells in G2/M phase gradually increased from 8.86% (0 μM) to 13.65% (0.1 μM), 18.37% (0.2 μM) and 26.42% (0.5 μM), while the G0/G1 phase cells decreased from 65.93% to 49.30%. These results suggested that compound 6j could arrest the cell cycle of SMMC-7721 cells at G2/M phase.

#### Tubulin polymerization inhibition

**Fig. 3:**
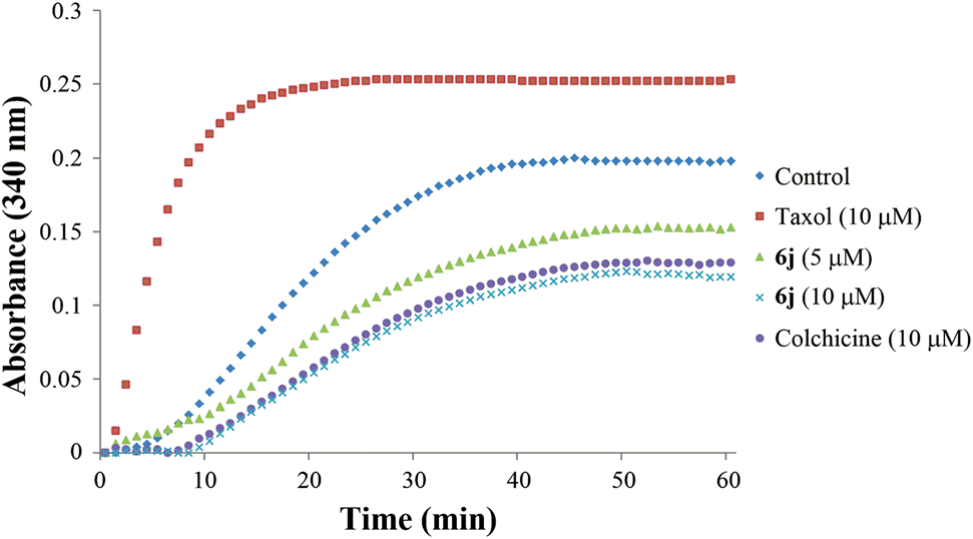
Effect of compound 6j on tubulin polymerization. Colchicine (10 μM) and taxol (10 μM) was used as the positive control (purple) and negative control (red), respectively, and G-PEM buffer was used as the blank control (blue). Tubulin was also treated with compound 6j at 5 μM (yellow) and 10 μM (green) concentrations. The reaction was monitored at OD 340 nm at 37 °C for 1 h.

The significant cell growth inhibitory activity of compound 6j in combination with its G2/M phase arresting property enlightened us to investigate its possible inhibition on tubulin polymerization. The *in vitro* tubulin polymerization inhibition assay was carried out by using the Tubulin Polymerization Assay Kit (Cytoskeleton, Inc., USA) according to the protocol in the manual. Porcine brain tubulin was incubated with compound 6j at concentrations of 5 and 10 μM. The positive control colchicine and negative control taxol were co-assayed at the concentration of 10 μM, and G-PEM buffer was used as the blank control. As shown in Fig. 3, taxol obviously increased the speed of tubulin polymerization and the final absorbance (OD_340_) compared with the blank control, indicating that it could enhance the tubulin polymerization. In contrast, compound 6j substantially decreased the polymerization speed and the final absorbance in a dose-dependent manner, and the inhibition potency of 6j was stronger than colchicine at 10 μM concentration. These results clearly indicate that compound 6j can significantly inhibit tubulin polymerization *in vitro*.

#### Immunofluorescence staining

**Fig. 4:**
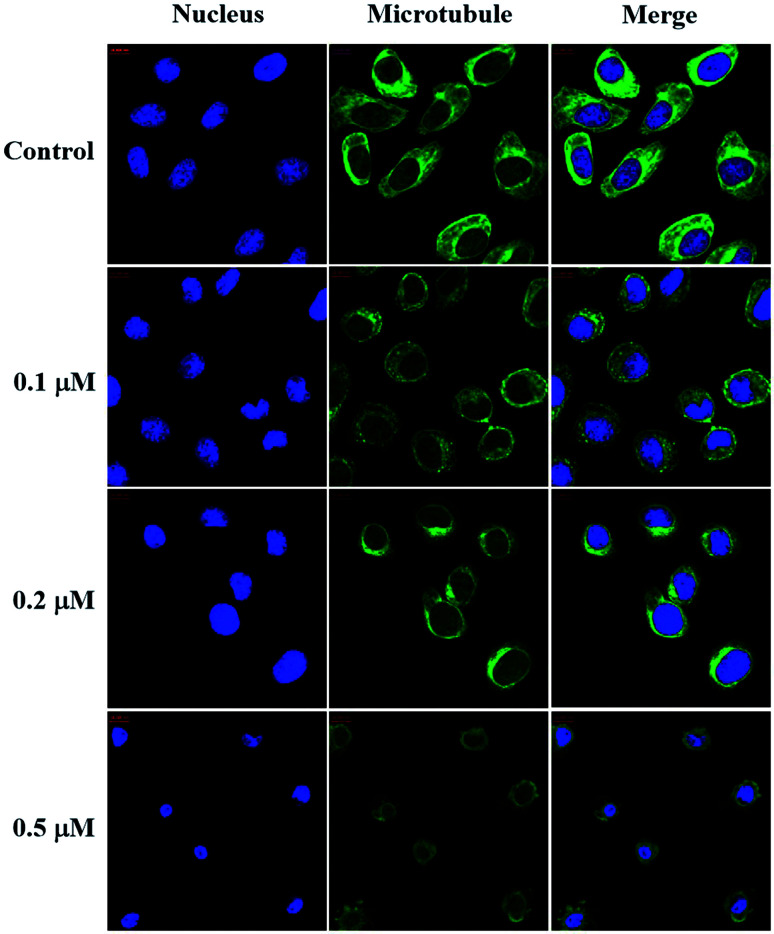
Effect of compound 6j on the microtubule network of SMMC-7721 cells. SMMC-7721 cells were untreated (control) and treated with compound 6j at 0.1, 0.2 and 0.5 μM for 24 h. Microtubules and unassembled tubulin were shown in green and the nuclei were in blue.

Since the tubulin polymerization inhibitory effect of compound 6j has been verified, we subsequently performed the immunofluorescent staining assay to observe its effect on the intracellular microtubule system of SMMC-7721 cells. As shown in Fig. 4, the nucleus and microtubules in the untreated group appeared as the normal state characterized by the slim and fibrous microtubules, well-organized structure (green) surrounding the uncondensed nuclei (blue). However, with increasing concentrations of compound 6j (0.1–0.5 μM), the meshy microtubule networks shrank around the nuclei in a dose-dependent manner. In 0.5 μM group, most microtubule networks disappeared, and most cell nuclei became irregularly shaped and highly condensed, which is a typical characteristic of cell apoptosis. These results indicated that compound 6j could disrupt microtubule organizations and therefore interfered with the mitosis of SMMC-7721 cells.

#### Wound healing assay

**Fig. 5:**
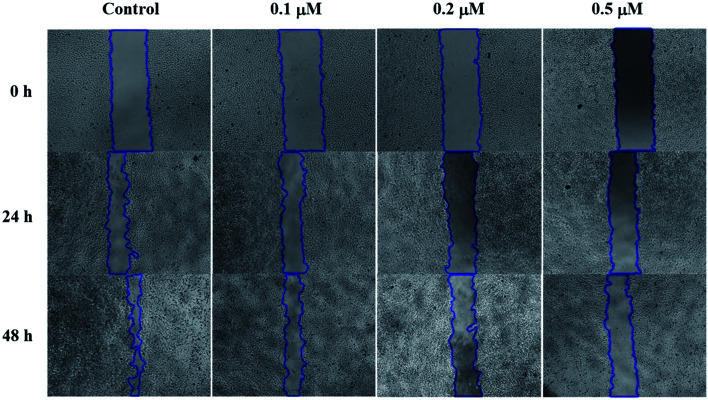
Effect of compound 6j on *in vitro* migration potential of SMMC-7721 cells. Scratches were created with sterile 200 μL pipette and images were captured using phase contrast microscopy at 0, 24 and 48 h after treatment with 0, 0.1, 0.2 and 0.5 μM of compound 6j.

Metastasis is a multistep process involving cancer cell motility and invasion which represents a key difficulty for cancer therapy. Thus, the inhibition of metastasis is vital for efficient cancer treatment. Microtubule plays an important role in cell motility and migration. As compound 6j inhibits tubulin polymerization, the capacity of migration inhibition was also investigated through wound healing assay.^45^ In this assay, standard scratches (wounds) were made in confluent monolayers of SMMC-7721 cells and incubated with different concentrations (0, 0.1, 0.2 and 0.5 μM) of compound 6j. The migration of SMMC-7721 cells was recorded by microscopic observations at 0, 24 and 48 h. As shown in Fig. 5, there was almost complete healing of wound in control group after 48 h, which indicated the considerable migration of cells. However, healing was strongly suppressed in 0.2 and 0.5 μM groups, suggesting that compound 6j could inhibit the migration of SMMC-7721 cells.

#### Cell apoptosis analysis

**Fig. 6:**
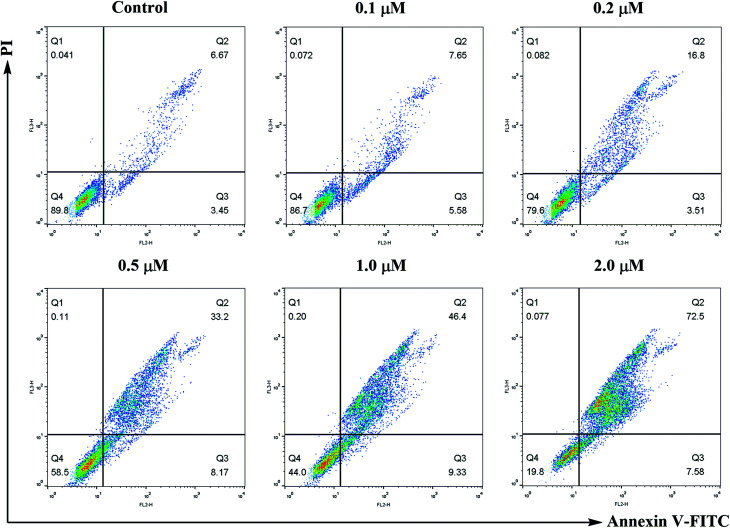
Annexin V-FITC/propidium iodide (PI) dual staining assay. SMMC-7721 cells were treated with compound 6j at 0, 0.1, 0.2, 0.5, 1.0 and 2.0 μM for 24 h, stained with Annexin V-FITC/PI and analyzed for apoptosis through flow cytometry. The percentage of cells positive for AV and/or PI is reported in the quadrants. Cells in the lower left quadrant (AV−/PI−): live cells; lower right quadrant (AV+/PI−): early apoptotic cells; upper right quadrant (AV+/PI+): late apoptotic cells; upper left quadrant (AV−/PI+): necrotic cells.

In order to investigate whether compound 6j could induce cell apoptosis, SMMC-7721 cells treated with different concentrations of compound 6j were subjected to Annexin V-FITC/propidium iodide (PI) dual staining followed by flow cytometry assay. As shown in Fig. 6, the percentage of early and late apoptotic cells (lower right quadrant, AV+/PI− and upper right quadrant, AV+/PI+, respectively) significantly increased from 10.12% to 80.08% after treatment with different concentrations of compound 6j (0, 0.1, 0.2, 0.5, 1.0 and 2.0 μM) for 24 h. The results suggested that compound 6j could induce the apoptosis of SMMC-7721 cells in a dose-dependent manner.

#### ROS generation assay

**Fig. 7:**
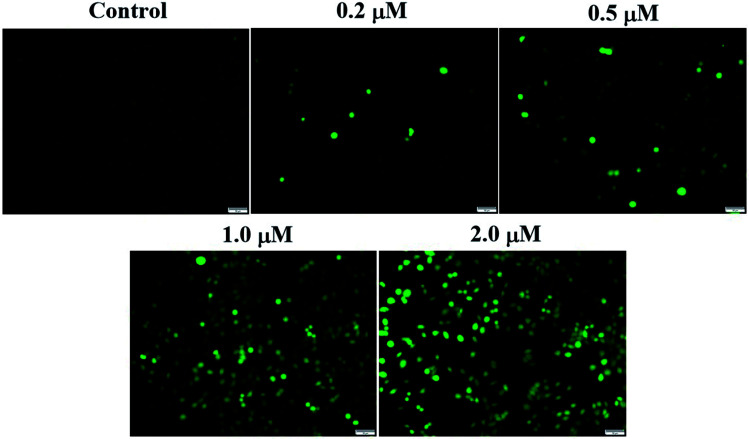
Effect of compound 6j on production of reactive oxygen species (ROS) in SMMC-7721 cells. SMMC-7721 cells were stained with DCF-DA after treatment with different concentrations of compound 6j (0, 0.2, 0.5, 1.0 and 2.0 μM) for 24 h. Fluorescent microscopic images of SMMC-7721 cells were acquired using fluorescence microscopy.

Reactive oxygen species (ROS) are highly harmful elements to cells as they exert oxidative stress and finally cause cellular damage. Excessive ROS generation renders cells vulnerable to apoptosis.^39^ To determine whether compound 6j triggers ROS generation in SMMC-7721 cells to induce apoptosis, the cells were treated with different concentrations (0, 0.2, 0.5, 1 and 2 μM) of compound 6j for 24 h, and the ROS generation was evaluated using the fluorescent probe 2,7-dichlorofluorescein diacetate (DCF-DA) by fluorescence microscopy. As shown in Fig. 7, SMMC-7721 cells treated with compound 6j appeared strong green fluorescence in a dose-dependent manner, indicating that compound 6j significantly induced ROS generation. This phenomenon implied that the increment of ROS might play an important role as an early mediator in compound 6j-induced apoptosis.

#### JC-1 mitochondrial membrane potential assay

**Fig. 8:**
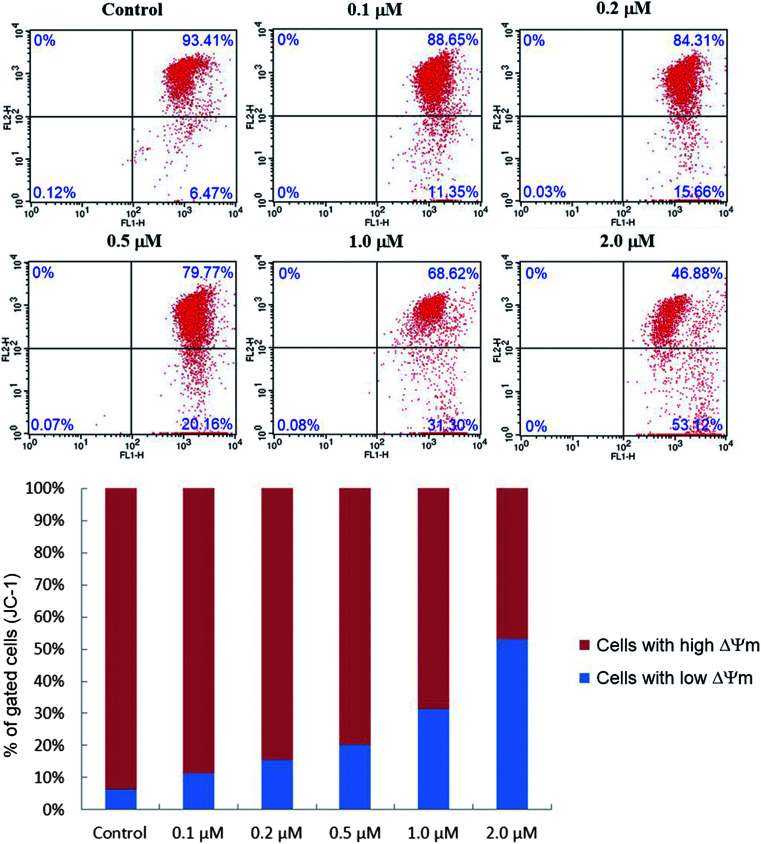
Effect of compound 6j on mitochondrial membrane potential (Δ*Ψ*_m_). SMMC-7721 cells were treated with different concentrations of compound 6j (0, 0.1, 0.2, 0.5, 1.0 and 2.0 μM), incubated with JC-1 and analyzed by flow cytometer.

As the most important organelle, mitochondria play key roles not only on supplying metabolic energy in the form of ATP, but also on regulating the signal transmission during the apoptosis of cancer cells.^46^ Besides, the mitochondrial dysfunction can be aroused under high ROS exposure, leading to the collapse of mitochondrial membrane potential (Δ*Ψ*_m_), which is a characteristic phenomenon of early apoptosis.^47^ In order to further investigate the apoptosis-inducing effect of target compound 6j, mitochondrial membrane potential changes were detected using the fluorescent probe JC-1. As a lipophilic cationic dye, JC-1 can easily pass through the plasma membrane into cells and accumulates in actively respiring mitochondria. In control cells, JC-1 can form aggregates in mitochondrial matrix and present high red fluorescence. However, in apoptotic cells whose mitochondrial potential has collapsed, JC-1 exists in cytosol as a monomer which emits green fluorescence. SMMC-7721 cells treated with different concentrations (0, 0.1, 0.2, 0.5, 1.0 and 2.0 μM) of compound 6j for 24 h were stained with JC-1 and then subjected to flow cytometry. The results were shown in Fig. 8.

As presented in Fig. 8, the percentage of cells with low Δ*Ψ*_m_ (lower right quadrant) increased from 6.47% (control) to 11.35% (0.1 μM), 15.66% (0.2 μM), 20.16% (0.5 μM), 31.30% (1.0 μM) and 53.12% (2.0 μM), which implied that compound 6j could cause the decrease of mitochondrial membrane potential in a dose dependent manner, and probably induced the apoptosis of SMMC-7721 cells through the intrinsic mitochondrial-mediated pathway.^48^

#### Molecular docking

In order to investigate the binding modes of dehydroabietic acid derivatives with the tubulin protein, the top active compound 6j and the reference ligand colchicine were selected and docked into the colchicine-binding site between α- and β-tubulin protein. (PDB ID: 1SA0).^49^ Molecular docking studies were performed by using GLIDE docking module of Schrödinger suite 2015-1.^50^

**Fig. 9:**
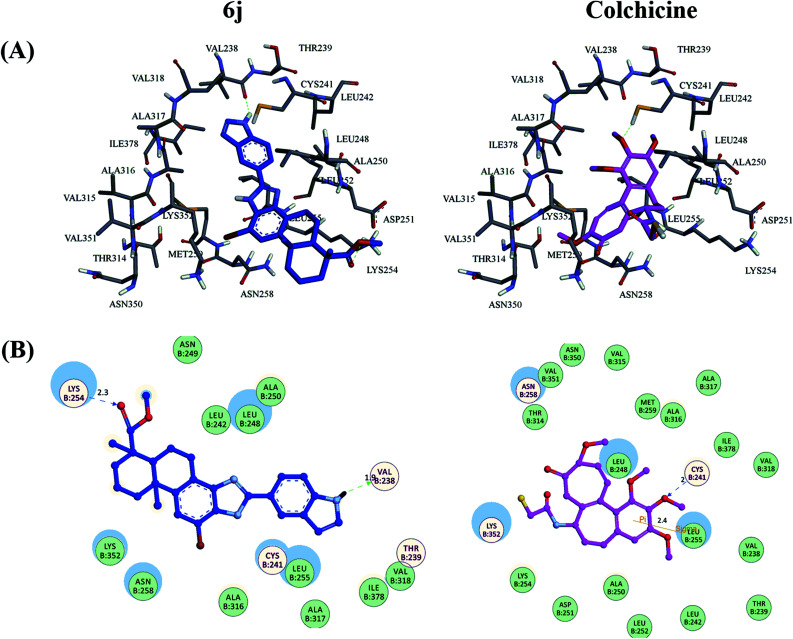
The binding modes between the active conformations of compound 6j, colchicine and tubulin. (A) The 3D diagram of interactions between the two compounds and the colchicine bing site. (B) The 2D diagram of interactions between the two compounds and the colchicine binding site. The H-bonds were displayed as dashed arrows, and the σ–π interactions were shown as orange lines.

As shown in Fig. 9(A), it was observed that the obtained binding pose of compound 6j could be well accommodated inside the colchicine-binding site of tubulin. The position of the imidazole moiety and dehydroabietic acid as a core scaffold of the derivative seemed substantially consistent in the active pocket, which was surmised that this series of the DAA derivatives would adopt similar kind of binding mode interacting with the tubulin protein. Fig. 9(B) illustrate the detailed protein-inhibitor interaction of compound 6j with key residues of the colchicine binding site. It could been observed that the N–H on the indole moiety of compound 6j established a hydrogen bond with the important active site residue Val B: 238, while the ester group of compound 6j also formed a hydrogen bond with Lys B: 254, which implied that the indole and methyl ester moieties played an important role in its anti-tubulin polymerization activity. In addition, the docking results showed that compound 6j displayed potent interaction energy (−8.888 kcal mol^−1^), superior to the docking score of colchicine (−6.557 kcal mol^−1^), which can further proved the effectiveness of this series of DAA derivatives targeting tubulin polymerization.

## Conclusion

In this work, a series of new benzimidazole derivatives of dehydroabietic acid were synthesized and evaluated for their *in vitro* anticancer potential against four cancer cell lines (SMMC-7721, MDA-MB-231, HeLa and CT-26). In MTT assay, compound 6j, 6k, 7j and 7k displayed significant growth inhibitory activities against several cancer cell lines comparable or superior to the positive control nocodazole. Among them, compound 6j exhibited the most potent anticancer activity against all test cancer cell lines and it was found to be comparatively safe towards human normal hepatocyte cell line QSG-7701. The treatment of SMMC-7721 cells with compound 6j led to inhibition of cell migration ability. Subsequently, the cell cycle analysis indicated that compound 6j could arrest SMMC-7721 cells at G2/M phase in a dose-dependent manner, which implied that compound 6j might interact with the microtubule systems of cancer cells. Tubulin polymerization inhibition assay and immunofluorescence staining further confirmed that compound 6j could considerably inhibit the polymerization of tubulin and thus disrupt the microtubule network of cancer cells. The molecular docking study provided insight into the binding mode of compound 6j in the colchicine-binding site of tubulin. On the other hand, Annexin V-FITC/PI dual staining assay suggested that compound 6j could induce the apoptosis of SMMC-7721 cells. The treatment with compound 6j also caused the collapse of mitochondrial membrane potential and enhanced intracellular ROS levels in SMMC-7721 cells. Therefore, compound 6j represented a novel lead for potent tubulin polymerization inhibitors that paved the way for the discovery of new promising anticancer agents.

## Experimental

### Chemistry

Melting points were measured on an XT-4 apparatus (Taike Corp., Beijing, China) and were uncorrected. IR spectra were measured on a Nexus 870 FT-IR spectrometer, and the absorption bands were expressed in cm^−1^. The ESI-MS spectra were recorded on a Mariner System 5304 mass spectrometer. Elemental analyses were carried out by Elementar Vario El cube elemental analyzer. ^1^H and ^13^C NMR spectra were accomplished in CDCl_3_ or DMSO-*d*_6_ on Bruker AV-300 and AV-500 NMR spectrometers using TMS as internal standard. Reactions and the resulted products were monitored by TLC which was carried out on TLC silica gel 60 F_254_ aluminium sheets from Merck KGaA, Darmstadt, Germany and visualized in UV light (254 nm). Silica gel (300–400 mesh) for column chromatography was purchased from Qingdao Marine Chemical Factory, China. The reagents (chemicals), all being of A.R. grade, were purchased from Shanghai Chemical Reagent Company (Shanghai, China) and Energy Chemical (Shanghai, China). Disproportionated rosin was provided by Zhongbang Chemicals Co., Ltd. (Zhaoqing, China), from which dehydroabietic acid (1, 97%) was isolated according to the published method.^51^

### General procedure for the synthesis of compounds 6a–k

The key intermediate (4) was synthesized from dehydroabietic acid (1) according to the procedure previously reported,^44^ which was further treated as follows to afford compounds 6a–k and 7a–k (Scheme 1). To a solution of compound 4 (0.44 g, 1.0 mmol) in 20 mL of EtOH was added reduced iron powder (0.6 g, 10.6 mmol), H_2_O (2 mL) and 10 drops of concentrated HCl. The mixture was stirred under reflux for 4 h. After cooling, the mixture was filtered to remove iron powder. The solution was neutralized with aqueous NaOH (2 mol L^−1^) and concentrated *in vacuo*. The residue was purified by column chromatography on silica gel, eluting with CH_2_Cl_2_–MeOH (20 : 1, v/v) to give 5 as a yellow resin (0.22 g, yield 57%). The spectral data of the product were in accordance with those in the previous literature.^44^

To a solution of compound 5 (0.66 g, 1.5 mmol) in 20 mL of anhydrous EtOH was added *p*-toluenesulfonic acid (25.8 mg, 0.15 mmol) and 2.2 mmol of different substituted arylaldehyde. The reaction mixture was stirred at 85 °C for 24 h under nitrogen atmosphere and monitored by TLC. At the end of reaction, the mixture was poured into 200 mL of ice-cold water, which was extracted with EtOAc for three times (3 × 200 mL). The organic phase was combined, washed with water, saturated NaHCO_3_ solution and brine, dried over anhydrous Na_2_SO_4_ and then concentrated *in vacuo* to give the crude product, which was purified by silica gel chromatography (petroleum ether–acetone 100 : 1–10 : 1, v/v) to afford compounds 6a–k.

#### Methyl 2′-phenyl-12-bromo-13,14-imidazolyl-deisopropyl-dehydroabietate (6a)

White powder solid; mp 123–125 °C; yield: 73.8%; ^1^H NMR (300 MHz, CDCl_3_) *δ*: 1.28 (s, 3H), 1.31 (s, 3H), 1.50–1.81 (m, 7H), 1.92 (m, 1H), 2.30–2.37 (m, 2H), 3.09 (m, 1H), 3.17 (brs, 1H), 3.68 (s, 3H), 7.35 (s, 1H), 7.45–7.48 (m, 3H), 8.04 (dd, *J* = 7.9, 1.8 Hz, 2H); ^13^C NMR (75 MHz, CDCl_3_) *δ*: 16.6, 18.7, 21.1, 25.3, 25.4, 36.8, 37.7, 38.6, 45.3, 47.9, 52.2, 104.7, 121.2, 122.5, 126.9, 129.0, 129.8, 130.3, 138.3, 139.8, 145.7, 151.5, 179.2; IR (KBr, cm^−1^): 3303, 2930, 2864, 1726, 1619, 1579, 1470, 1454, 1385, 1254, 1193, 1129, 1035, 857, 737; MS (ESI): *m*/*z* 467.1 [M + H]^+^; anal. calcd for C_25_H_27_BrN_2_O_2_: C 64.24; H 5.82; N 5.99; found: C 64.31; H 5.78, N 6.03.

#### Methyl 2′-(*p*-tolyl)-12-bromo-13,14-imidazolyl-deisopropyl-dehydroabietate (6b)

White powder solid; mp 143–145 °C; yield: 71.6%; ^1^H NMR (500 MHz, CDCl_3_) *δ*: 1.27 (s, 3H), 1.31 (s, 3H), 1.50–1.81 (m, 7H), 1.92 (m, 1H), 2.30–2.36 (m, 2H), 2.41 (s, 3H), 3.07 (brs, 1H), 3.17 (brs, 1H), 3.68 (s, 3H), 7.28 (d, *J* = 7.9 Hz, 2H), 7.33 (s, 1H), 7.93 (d, *J* = 8.0 Hz, 2H); ^13^C NMR (75 MHz, CDCl_3_) *δ*: 16.6, 18.7, 21.1, 21.6, 25.4, 36.8, 37.6, 38.6, 45.2, 47.9, 52.2, 104.6, 122.4, 126.8, 126.9, 129.8, 134.1, 139.2, 140.7, 145.6, 151.7, 179.2; IR (KBr, cm^−1^): 3308, 2929, 2867, 1725, 1623, 1579, 1476, 1429, 1380, 1253, 1190, 1128, 1037, 824, 726; MS (ESI): *m*/*z* 481.1 [M + H]^+^; anal. calcd for C_26_H_29_BrN_2_O_2_: C 64.87; H 6.07; N 5.82; found: C 64.81; H 6.13, N 5.76.

#### Methyl 2′-(4-fluorophenyl)-12-bromo-13,14-imidazolyl-deisopropyl-dehydroabietate (6c)

White powder solid; mp 149–151 °C; yield: 82.6%; ^1^H NMR (500 MHz, CDCl_3_) *δ*: 1.28 (s, 3H), 1.32 (s, 3H), 1.50–1.81 (m, 7H), 1.92 (m, 1H), 2.30–2.36 (m, 2H), 3.06 (brs, 1H), 3.17 (brs, 1H), 3.68 (s, 3H), 7.16 (t, *J* = 8.5 Hz, 2H), 7.35 (s, 1H), 8.02 (dd, *J* = 8.5, 5.2 Hz, 2H); ^13^C NMR (75 MHz, CDCl_3_) *δ*: 16.6, 18.7, 21.1, 25.3, 25.4, 36.8, 37.7, 38.6, 45.2, 47.9, 52.2, 103.0, 116.2 (d, *J* = 21.9 Hz), 122.6, 126.1, 128.9 (d, *J* = 8.5 Hz), 139.2, 140.6, 145.9, 150.6, 164.1 (d, *J* = 249.1 Hz), 179.3; IR (KBr, cm^−1^): 3313, 2931, 2863, 1729, 1620, 1607, 1493, 1471, 1431, 1381, 1250, 1160, 1128, 841, 736; MS (ESI): *m*/*z* 485.1 [M + H]^+^; anal. calcd for C_25_H_26_BrFN_2_O_2_: C 61.86; H 5.40; N 5.77; found: C 61.84; H 5.42, N 5.78.

#### Methyl 2′-(3-fluorophenyl)-12-bromo-13,14-imidazolyl-deisopropyl-dehydroabietate (6d)

Yellow powder solid; mp 142–144 °C; yield: 74.2%; ^1^H NMR (300 MHz, CDCl_3_) *δ*: 1.28 (s, 3H), 1.32 (s, 3H), 1.50–1.82 (m, 7H), 1.93 (m, 1H), 2.30–2.36 (m, 2H), 3.07 (m, 1H), 3.16 (m, 1H), 3.69 (s, 3H), 7.16 (t, *J* = 8.4 Hz, 1H), 7.36 (s, 1H), 7.46 (m, 1H), 7.79 (d, *J* = 8.1 Hz, 2H); ^13^C NMR (75 MHz, CDCl_3_) *δ*: 16.6, 18.7, 21.1, 25.3, 25.4, 36.8, 37.7, 38.6, 45.3, 47.9, 52.2, 105.9, 114.1 (d, *J* = 23.4 Hz), 117.3 (d, *J* = 21.2 Hz), 122.4 (d, *J* = 2.8 Hz), 122.9, 127.3 (d, *J* = 7.1 Hz), 130.8 (d, *J* = 8.2 Hz), 136.1, 140.2, 145.2, 147.9, 164.1 (d, *J* = 249.1 Hz), 179.3; IR (KBr, cm^−1^): 3285, 2926, 2854, 1724, 1665, 1586, 1461, 1255, 1198, 1129, 861, 790; MS (ESI): *m*/*z* 485.1 [M + H]^+^; anal. calcd for C_25_H_26_BrFN_2_O_2_: C 61.86; H 5.40; N 5.77; found: C 61.93; H 5.36, N 5.71.

#### Methyl 2′-(2-fluorophenyl)-12-bromo-13,14-imidazolyl-deisopropyl-dehydroabietate (6e)

Yellow powder solid; mp 138–139 °C; yield: 72.7%; ^1^H NMR (300 MHz, CDCl_3_) *δ*: 1.28 (s, 3H), 1.32 (s, 3H), 1.50–1.82 (m, 7H), 1.95 (m, 1H), 2.31–2.37 (m, 2H), 3.07–3.16 (m, 2H), 3.69 (s, 3H), 7.20 (m, 1H), 7.29–7.34 (m, 2H), 7.40–7.48 (m, 2H); ^13^C NMR (75 MHz, CDCl_3_) *δ*: 16.6, 18.7, 21.1, 25.3, 36.7, 37.7, 38.6, 45.3, 47.8, 52.2, 106.4, 116.2 (d, *J* = 23.0 Hz), 122.8, 125.3 (d, *J* = 2.9 Hz), 130.8 (d, *J* = 23.8 Hz), 131.8 (d, *J* = 9.1 Hz), 137.8, 139.8, 145.9, 151.8, 160.4 (d, *J* = 245.6 Hz), 179.1; IR (KBr, cm^−1^): 3379, 2921, 2780, 2360, 1712, 1455, 1245, 1128, 1045, 880, 758, 692. MS (ESI): *m*/*z* 486.4 [M + H]^+^; anal. calcd (%) for C_25_H_26_BrFN_2_O_2_: C, 61.86; H, 5.40; N, 5.77; found (%): C, 61.92; H, 5.37; N, 5.82.

#### Methyl 2′-(4-chlorophenyl)-12-bromo-13,14-imidazolyl-deisopropyl-dehydroabietate (6f)

Yellow powder solid; mp 139–141 °C; yield: 81.8%; ^1^H NMR (300 MHz, CDCl_3_) *δ*: 1.28 (s, 3H), 1.32 (s, 3H), 1.50–2.10 (m, 7H), 1.93 (m, 1H), 2.30–2.37 (m, 2H), 3.05–3.16 (m, 2H), 3.68 (s, 3H), 7.36 (s, 1H), 7.46 (d, *J* = 8.1 Hz, 2H), 7.98 (d, *J* = 8.3 Hz, 2H); ^13^C NMR (75 MHz, CDCl_3_) *δ*: 16.6, 18.7, 21.1, 25.3, 25.4, 36.8, 37.7, 38.5, 45.2, 47.9, 52.2, 105.1, 122.8, 128.1, 128.3, 129.3, 136.4, 139.7, 141.3, 146.0, 150.3, 179.3; IR (KBr, cm^−1^): 3355, 2924, 2852, 1724, 1666, 1577, 1494, 1454, 1244, 1126, 1046, 1015, 764, 732. MS (ESI): *m*/*z* 501.1 [M + H]^+^; anal. calcd (%) for C_25_H_26_BrClN_2_O_2_: C, 59.83; H, 5.22; N, 5.58; found (%): C, 59.76; H, 5.27; N, 5.62.

#### Methyl 2′-(2-chlorophenyl)-12-bromo-13,14-imidazolyl-deisopropyl-dehydroabietate (6g)

Yellow powder solid; mp 142–143 °C; yield: 75.8%; ^1^H NMR (300 MHz, CDCl_3_) *δ*: 1.29 (s, 3H), 1.32 (s, 3H), 1.50–2.10 (m, 7H), 1.94 (m, 1H), 2.30–2.42 (m, 2H), 3.02–3.30 (m, 2H), 3.69 (s, 3H), 7.39 (s, 1H), 7.41–7.45 (m, 2H), 7.49 (dd, *J* = 7.2, 1.8 Hz, 1H), 8.43 (d, *J* = 7.2 Hz, 1H); ^13^C NMR (75 MHz, CDCl_3_) *δ*: 16.6, 18.7, 21.0, 25.34, 25.36, 36.7, 37.7, 38.6, 45.2, 47.8, 52.1, 105.8, 122.8, 127.6, 128.4, 129.0, 130.6, 131.1, 132.5, 138.7, 141.1, 145.8, 150.4, 179.1; IR (KBr, cm^−1^): 3301, 2929, 2866, 1725, 1617, 1578, 1537, 1451, 1404, 1250, 1192, 1129, 1052, 1035, 962, 764, 735. MS (ESI): *m*/*z* 501.1 [M + H]^+^; anal. calcd (%) for C_25_H_26_BrClN_2_O_2_: C, 59.83; H, 5.22; N, 5.58; found (%): C, 59.88; H, 5.29; N, 5.51.

#### Methyl 2′-(4-bromophenyl)-12-bromo-13,14-imidazolyl-deisopropyl-dehydroabietate (6h)

Yellow powder solid; mp 141–142 °C; yield: 74.2%; ^1^H NMR (300 MHz, CDCl_3_) *δ*: 1.28 (s, 3H), 1.32 (s, 3H), 1.50–2.10 (m, 8H), 2.30–2.37 (m, 2H), 3.05–3.17 (m, 2H), 3.68 (s, 3H), 7.36 (s, 1H), 7.61 (d, *J* = 8.2 Hz, 2H), 7.92 (d, *J* = 8.3 Hz, 2H); ^13^C NMR (75 MHz, CDCl_3_) *δ*: 16.6, 18.7, 21.1, 25.4, 25.5, 36.8, 37.7, 38.5, 45.2, 47.9, 52.2, 105.2, 122.8, 124.9, 128.4, 129.0, 131.1, 132.2, 136.9, 139.2, 145.2, 150.5, 179.3; IR (KBr, cm^−1^): 3317, 2923, 2852, 1725, 1468, 1428, 1382, 1254, 1191, 1129, 1072, 1038, 1010, 834, 727. MS (ESI): *m*/*z* 545.0 [M + H]^+^; anal. calcd (%) for C_25_H_26_Br_2_N_2_O_2_: C, 54.96; H, 4.80; N, 5.13; found (%): C, 55.03; H, 4.84; N, 5.08.

#### Methyl 2′-(4-methoxyphenyl)-12-bromo-13,14-imidazolyl-deisopropyl-dehydroabietate (6i)

White powder solid; mp 140–142 °C; yield: 61.3%; ^1^H NMR (500 MHz, CDCl_3_) *δ*: 1.27 (s, 3H), 1.31 (s, 3H), 1.50–1.81 (m, 7H), 1.91 (m, 1H), 2.33 (m, 2H), 3.03 (m, 1H), 3.17 (brs, 1H), 3.67 (s, 3H), 3.86 (s, 3H), 6.98 (d, *J* = 8.5 Hz, 2H), 7.31 (s, 1H), 7.98 (d, *J* = 8.4 Hz, 2H); ^13^C NMR (75 MHz, CDCl_3_) *δ*: 16.6, 18.7, 21.1, 25.3, 25.4, 36.8, 37.6, 38.5, 45.2, 47.8, 52.2, 55.5, 105.9, 114.4, 122.2, 122.4, 128.5, 136.2, 138.7, 145.4, 151.9, 161.3, 179.3; IR (KBr, cm^−1^): 3331, 2934, 2863, 1724, 1613, 1581, 1496, 1474, 1435, 1385, 1254, 1178, 1129, 1033, 838, 737; MS (ESI): *m*/*z* [M + H]^+^: 497.1; anal. calcd for C_26_H_29_BrN_2_O_3_: C 62.78; H 5.88; N 5.63; found: C 62.70; H 5.93, N 5.67.

#### Methyl 2′-(1*H*-indol-5-yl)-12-bromo-13,14-imidazolyl-deisopropyl-dehydroabietate (6j)

White powder solid; mp 179–181 °C; yield: 74.4%; ^1^H NMR (500 MHz, CDCl_3_) *δ*: 1.28 (s, 3H), 1.32 (s, 3H), 1.50–1.82 (m, 7H), 1.94 (m, 1H), 2.31–2.37 (m, 2H), 3.09 (m, 1H), 3.21 (brs, 1H), 3.69 (s, 3H), 6.62 (s, 1H), 7.28 (s, 1H), 7.32 (s, 1H), 7.45 (d, *J* = 8.1 Hz, 1H), 7.90 (d, *J* = 8.2 Hz, 1H), 8.29 (s, 1H), 8.44 (brs, 1H); ^13^C NMR (75 MHz, CDCl_3_) *δ*: 16.6, 18.7, 21.1, 25.3, 25.4, 36.8, 37.5, 38.5, 45.2, 47.8, 52.2, 103.1, 107.6, 111.8, 116.3, 119.8, 121.1, 121.2, 121.9, 125.8, 128.0, 136.2, 137.0, 139.8, 145.2, 153.9, 179.5; IR (KBr, cm^−1^): 3325, 2929, 2866, 1703, 1620, 1581, 1542, 1452, 1397, 1250, 1130, 1035, 811, 735; MS (ESI): *m*/*z* 506.1 [M + H]^+^; anal. calcd for C_27_H_28_BrN_3_O_2_: C 64.03; H 5.57; N 8.30; found: C 64.09; H 5.53, N 8.37.

#### Methyl 2′-(1*H*-indol-3-yl)-12-bromo-13,14-imidazolyl-deisopropyl-dehydroabietate (6k)

White powder solid; mp 139–140 °C; yield: 60.2%; ^1^H NMR (300 MHz, DMSO-*d*_6_) *δ*: 1.23 (s, 3H), 1.25 (s, 3H), 1.36–1.51 (m, 2H), 1.63–2.00 (m, 5H), 2.15–2.39 (m, 2H), 2.37 (m, 1H), 2.90 (m, 1H), 3.06 (m, 1H), 3.64 (s, 3H), 7.23 (m, 3H), 7.48 (d, *J* = 5.2 Hz, 1H), 8.25 (s, 1H), 8.56 (s, 1H), 11.60 (s, 1H); ^13^C NMR (75 MHz, CDCl_3_) *δ*: 16.6, 18.7, 21.1, 25.4, 36.8, 37.6, 38.6, 45.2, 47.8, 52.2, 106.0, 106.9, 112.2, 120.1, 121.4, 121.8, 123.1, 125.1, 126.1, 129.7, 135.7, 136.5, 138.5, 145.3, 149.4, 179.3; IR (KBr, cm^−1^): 3393, 3327, 2929, 2870, 1724, 1705, 1627, 1587, 1542, 1456, 1390, 1247, 1129, 1023, 821, 743; MS (ESI): *m*/*z* 506.1 [M + H]^+^; anal. calcd for C_27_H_28_BrN_3_O_2_: C 64.03; H 5.57; N 8.30; found: C 63.95; H 5.64, N 8.26.

### General procedure for the synthesis of compounds 7a–k

To a solution of compound 6 (0.15 mmol) in 22 mL of THF/H_2_O (10 : 1) was added 3,4,5-trimethoxyphenylboronic acid (63 mg, 0.3 mmol), K_2_CO_3_ (415 mg, 3 mmol) and Pd(PPh_3_)_4_ (17 mg, 0.015 mmol). The reaction mixture was stirred at 85 °C for 24 h under nitrogen atmosphere and monitored by TLC. Subsequently, the mixture was poured into ice-cold water (200 mL) and extracted EtOAc for three times (3 × 200 mL). The organic layer was combined, washed with water, saturated NaHCO_3_ solution and brine, dried over anhydrous Na_2_SO_4_ and then concentrated *in vacuo* to give the crude product, which was purified by silica gel chromatography (petroleum ether-acetone 100 : 1–10 : 1, v/v) to afford compounds 7a–k.

#### Methyl 2′-phenyl-12-(3,4,5-trimethoxyphenyl)-13,14-imidazolyl-deisopropyl-dehydroabietate (7a)

White powder solid; mp 153–155 °C; yield: 62.5%; ^1^H NMR (500 MHz, CDCl_3_) *δ*: 1.35 (s, 6H), 1.50–1.82 (m, 7H), 2.00 (m, 1H), 2.42 (d, *J* = 12.4 Hz, 1H), 2.47 (m, 1H), 3.07 (brs, 1H), 3.14 (m, 1H), 3.70 (s, 3H), 3.93 (s, 6H), 3.96 (s, 3H), 6.80 (s, 2H), 7.36 (s, 1H), 7.40–7.49 (m, 3H), 8.02 (brs, 2H); ^13^C NMR (75 MHz, CDCl_3_) *δ*: 16.7, 18.8, 21.3, 25.5, 25.6, 36.8, 37.6, 38.7, 45.5, 48.0, 52.1, 56.5, 61.1, 106.2, 118.9, 119.8, 126.2, 126.6, 128.8, 129.1, 130.1, 134.9, 137.6, 140.3, 140.7, 147.6, 151.1, 153.7, 179.2; IR (KBr, cm^−1^): 3318, 2935, 2870, 1724, 1581, 1496, 1454, 1378, 1246, 1180, 1128, 1023, 833, 735; MS (ESI): *m*/*z* 555.3 [M + H]^+^; anal. calcd for C_34_H_38_N_2_O_5_: C 73.62; H 6.91; N 5.05; found: C 73.69; H 6.88, N 5.10.

#### Methyl 2′-(*p*-tolyl)-12-(3,4,5-trimethoxyphenyl)-13,14-imidazolyl-deisopropyl-dehydroabietate (7b)

White powder solid; mp 133–135 °C; yield: 56.3%; ^1^H NMR (500 MHz, CDCl_3_) *δ*: 1.26 (s, 3H), 1.34 (s, 3H), 1.50–1.86 (m, 7H), 2.01 (m, 1H), 2.41 (s, 3H), 2.45 (m, 2H), 3.19 (brs, 1H), 3.28 (brs, 1H), 3.69 (s, 3H), 3.93 (s, 3H), 3.95 (s, 6H), 6.82 (s, 2H), 7.29 (d, *J* = 7.9 Hz, 2H), 7.37 (s, 1H), 7.92 (d, *J* = 7.4 Hz, 2H); ^13^C NMR (75 MHz, CDCl_3_) *δ*: 16.7, 18.8, 21.3, 21.6, 25.5, 25.6, 36.8, 37.6, 38.7, 45.4, 47.9, 52.1, 56.5, 61.1, 106.2, 119.0, 119.7, 126.8, 127.0, 128.6, 128.8, 129.8, 132.2, 137.8, 140.0, 140.5, 144.6, 151.3, 153.6, 179.2; IR (KBr, cm^−1^): 3330, 2925, 2854, 1725, 1666, 1581, 1497, 1459, 1382, 1245, 1186, 1127, 1021, 826, 731; MS (ESI): *m*/*z* 569.3 [M + H]^+^; anal. calcd for C_35_H_40_N_2_O_5_: C 73.92; H 7.09; N 4.93; found: C 73.87; H 7.13, N 4.99.

#### Methyl 2′-(4-fluorophenyl)-12-(3,4,5-trimethoxyphenyl)-13,14-imidazolyl-deisopropyl-dehydroabietate (7c)

White powder solid; mp 139–141 °C; yield: 50.1%; ^1^H NMR (500 MHz, CDCl_3_) *δ*: 1.26 (s, 3H), 1.34 (s, 3H), 1.60–1.85 (m, 7H), 2.01 (m, 1H), 2.41 (d, *J* = 12.3 Hz, 1H), 2.46 (d, *J* = 13.7 Hz, 1H), 3.17 (brs, 1H), 3.26 (brs, 1H), 3.70 (s, 3H), 3.92 (s, 3H), 3.95 (s, 6H), 6.84 (s, 2H), 7.16 (t, *J* = 8.3 Hz, 2H), 7.36 (s, 1H), 8.03 (brs, 2H); ^13^C NMR (75 MHz, CDCl_3_) *δ*: 16.7, 18.8, 21.3, 25.5, 25.6, 36.9, 37.6, 38.7, 45.5, 48.0, 52.1, 56.5, 61.1, 106.3, 116.2 (d, *J* = 21.9 Hz), 119.2, 119.3, 123.6, 124.1, 124.5, 124.6, 128.6 (d, *J* = 8.5 Hz), 137.7, 140.1, 147.3, 153.6, 154.4, 164.0 (d, *J* = 249.8 Hz), 179.3; IR (KBr, cm^−1^): 3308, 2959, 2925, 2855, 1726, 1598, 1579, 1493, 1433, 1387, 1245, 1158, 1128, 1021, 843, 735; MS (ESI): *m*/*z* 573.3 [M + H]^+^; anal. calcd for C_34_H_37_FN_2_O_5_: C 71.31; H 6.51; N 4.89; found: C 71.38; H 6.48, N 4.83.

#### Methyl 2′-(3-fluorophenyl)-12-(3,4,5-trimethoxyphenyl)-13,14-imidazolyl-deisopropyl-dehydroabietate (7d)

Yellow powder solid; mp 156–158 °C; yield: 72.9%; ^1^H NMR (300 MHz, CDCl_3_) *δ*: 1.26 (s, 3H), 1.35 (s, 3H), 1.50–1.90 (m, 7H), 2.00 (m, 1H), 2.42 (d, *J* = 12.5 Hz, 1H), 2.49 (m, 1H), 3.10 (m, 1H), 3.22 (m, 1H), 3.70 (s, 3H), 3.92 (s, 3H), 3.95 (s, 6H), 6.82 (s, 2H), 7.14 (t, *J* = 7.6 Hz, 1H), 7.35 (s, 1H), 7.45 (m, 1H), 7.77 (d, *J* = 8.3 Hz, 2H); ^13^C NMR (125 MHz, CDCl_3_) *δ*: 16.7, 18.8, 21.3, 25.4, 25.6, 36.9, 37.7, 38.6, 45.5, 48.0, 52.2, 56.5, 61.1, 106.3, 113.8 (d, *J* = 23.5 Hz), 116.9 (d, *J* = 20.9 Hz), 119.2, 119.5, 122.2, 124.6, 130.7 (d, *J* = 8.2 Hz), 132.5 (d, *J* = 8.4 Hz), 134.7, 136.7, 137.9, 139.6, 144.9, 149.9, 153.6, 163.2 (d, *J* = 245.2 Hz), 179.4; IR (KBr, cm^−1^): 3305, 2928, 2854, 1724, 1659, 1582, 1492, 1462, 1243, 1126, 1006, 790, 760; MS (ESI): *m*/*z* 573.3 [M + H]^+^; anal. calcd for C_34_H_37_FN_2_O_5_: C 71.31; H 6.51; N 4.89; found: C 71.22; H 6.57, N 4.92.

#### Methyl 2′-(2-fluorophenyl)-12-(3,4,5-trimethoxyphenyl)-13,14-imidazolyl-deisopropyl-dehydroabietate (7e)

Yellow powder solid; mp 155–157 °C; yield: 53.1%; ^1^H NMR (300 MHz, CDCl_3_) *δ*: 1.35 (s, 6H), 1.51–1.90 (m, 7H), 2.01 (m, 1H), 2.42 (d, *J* = 11.7 Hz, 1H), 2.48 (m, 1H), 3.07 (brs, 1H), 3.30 (m, 1H), 3.69 (s, 3H), 3.95 (s, 6H), 3.99 (s, 3H), 6.84 (s, 2H), 7.20 (m, 1H), 7.31–7.43 (m, 4H); ^13^C NMR (75 MHz, CDCl_3_) *δ*: 16.7, 18.8, 21.3, 25.4, 25.5, 36.8, 37.7, 38.7, 45.5, 48.0, 52.1, 56.5, 61.1, 107.5, 116.2 (d, *J* = 22.8 Hz), 117.8 (d, *J* = 9.9 Hz), 118.6, 119.5, 123.4 (d, *J* = 24.9 Hz), 125.3, 130.4, 131.4 (d, *J* = 6.8 Hz), 134.7, 137.8, 136.6, 140.0, 146.6, 152.8, 153.9, 160.4 (d, *J* = 245.0 Hz), 179.2; IR (KBr, cm^−1^): 3353, 2928, 2867, 2280, 1723, 1581, 1494, 1464, 1245, 1178, 1065, 1015, 824, 761. MS (ESI): *m*/*z* 573.3 [M + H]^+^; anal. calcd for C_34_H_37_FN_2_O_5_: C 71.31; H 6.51; N 4.89; found: C 71.36; H 6.59, N 4.81.

#### Methyl 2′-(4-chlorophenyl)-12-(3,4,5-trimethoxyphenyl)-13,14-imidazolyl-deisopropyl-dehydroabietate (7f)

Yellow powder solid; mp 168–170 °C; yield: 59.9%; ^1^H NMR (300 MHz, CDCl_3_) *δ*: 1.26 (s, 3H), 1.35 (s, 3H), 1.50–1.85 (m, 7H), 1.99 (m, 1H), 2.41 (d, *J* = 12.5 Hz, 1H), 2.48 (m, 1H), 3.16 (brs, 1H), 3,23 (brs, 1H), 3.69 (s, 3H), 3.92 (s, 3H), 3.95 (s, 6H), 6.82 (s, 2H), 7.35 (s, 1H), 7.46 (d, *J* = 8.4 Hz, 2H), 7.97 (d, *J* = 7.7 Hz, 2H); ^13^C NMR (75 MHz, CDCl_3_) *δ*: 16.7, 18.8, 21.3, 25.4, 25.6, 36.9, 37.6, 38.6, 45.5, 48.0, 52.2, 56.5, 61.1, 106.3, 119.0, 119.2, 124.5, 127.9, 128.8, 129.3, 134.8, 136.0, 137.8, 139.0, 140.8, 141.3, 150.1, 153.6, 179.4; IR (KBr, cm^−1^): 3317, 2926, 2853, 1725, 1581, 1495, 1461, 1428, 1381, 1244, 1127, 1014, 836, 730. MS (ESI): *m*/*z* 589.2 [M + H]^+^; anal. calcd (%) for C_34_H_37_ClN_2_O_5_: C, 69.32; H, 6.33; N, 4.76; found (%): C, 69.38; H, 6.25; N, 4.82.

#### Methyl 2′-(2-chlorophenyl)-12-(3,4,5-trimethoxyphenyl)-13,14-imidazolyl-deisopropyl-dehydroabietate (7g)

Yellow powder solid; mp 157–159 °C; yield: 72.4%; ^1^H NMR (300 MHz, CDCl_3_) *δ*: 1.26 (s, 3H), 1.35 (s, 3H), 1.50–2.10 (m, 7H), 2.42 (d, *J* = 11.7 Hz, 1H), 2.48 (m, 1H), 3.06 (brs, 1H), 3.31 (m, 1H), 3.50 (m, 1H), 3.69 (s, 3H), 3.93 (s, 3H), 3.96 (s, 6H), 6.85 (s, 2H), 7.35–7.43 (m, 3H), 7.48 (d, *J* = 7.6 Hz, 1H), 8.44 (brs, 1H); ^13^C NMR (75 MHz, CDCl_3_) *δ*: 16.7, 18.8, 21.3, 25.4, 25.6, 36.8, 37.7, 38.8, 45.5, 48.0, 52.1, 56.4, 61.1, 106.9, 119.5, 119.7, 126.7, 127.7, 128.8, 129.5, 130.0, 130.7, 132.4, 133.2, 137.8, 139.5, 140.3, 146.1, 148.5, 153.4, 179.2; IR (KBr, cm^−1^): 3355, 2924, 2852, 1724, 1666, 1577, 1494, 1454, 1244, 1126, 1046, 1016, 764, 732. MS (ESI): *m*/*z* 589.2 [M + H]^+^; anal. calcd (%) for C_34_H_37_ClN_2_O_5_: C, 69.32; H, 6.33; N, 4.76; found (%): C, 69.26; H, 6.37; N, 4.71.

#### Methyl 2′-(4-bromophenyl)-12-(3,4,5-trimethoxyphenyl)-13,14-imidazolyl-deisopropyl-dehydroabietate (7h)

Yellow powder solid; mp 150–152 °C; yield: 54.1%; ^1^HNMR (300 MHz, CDCl_3_) *δ*: 1.26 (s, 3H), 1.35 (s, 3H), 1.50–1.90 (m, 7H), 2.00 (m, 1H), 2.42 (d, *J* = 11.8 Hz, 1H), 2.48 (m, 1H), 3.08 (brs, 1H), 3.28 (brs, 1H), 3.69 (s, 3H), 3.92 (s, 3H), 3.95 (s, 6H), 6.82 (s, 2H), 7.36 (s, 1H), 7.67 (d, *J* = 8.2 Hz, 2H), 8.10 (d, *J* = 9.3 Hz, 2H); ^13^C NMR (75 MHz, CDCl_3_) *δ*: 16.7, 18.8, 21.3, 25.4, 25.6, 36.8, 37.6, 38.6, 45.5, 48.0, 52.1, 56.4, 61.1, 104.7, 118.4, 119.7, 127.0, 127.6, 129.0, 129.2, 131.0, 136.2, 137.8, 138.4, 142.8, 144.5, 150.9, 153.8, 179.3; IR (KBr, cm^−1^): 3285, 2928, 2854, 1724, 1584, 1459, 1343, 1245, 1188, 1093, 1008, 878, 825, 745. MS (ESI): *m*/*z* 632.2, 634.2 [M + H]^+^; anal. calcd (%) for C_34_H_37_BrN_2_O_5_: C, 64.45; H, 5.89; N, 4.42; found (%): C, 64.36; H, 5.92; N, 4.38.

#### Methyl 2′-(4-methoxyphenyl)-12-(3,4,5-trimethoxyphenyl)-13,14-imidazolyl-deisopropyl-dehydroabietate (7i)

White powder solid; mp 153–155 °C; yield: 70.7%; ^1^H NMR (500 MHz, CDCl_3_) *δ*: 1.26 (s, 3H), 1.34 (s, 3H), 1.50–1.84 (m, 7H), 2.01 (m, 1H), 2.40 (d, *J* = 12.2 Hz, 2H), 2.46 (brs, 1H), 3.30 (m, 1H), 3.69 (s, 3H), 3.87 (s, 3H), 3.92 (s, 6H), 3.95 (s, 3H), 6.80 (s, 2H), 7.00 (d, *J* = 8.2 Hz, 2H), 7.35 (s, 1H), 7.96 (d, *J* = 8.2 Hz, 2H); ^13^C NMR (75 MHz, CDCl_3_) *δ*: 16.7, 18.8, 21.3, 25.5, 25.6, 36.8, 37.6, 38.7, 45.5, 48.0, 52.1, 55.6, 56.5, 61.1, 107.3, 113.9, 114.2, 114.5, 119.7, 121.5, 126.4, 128.1, 131.0, 136.7, 138.6, 140.6, 151.4, 153.2, 162.9, 179.3; IR (KBr, cm^−1^): 3330, 2925, 2854, 1725, 1666, 1581, 1497, 1459, 1382, 1245, 1186, 1127, 1021, 826, 731; MS (ESI): *m*/*z* 584.3 [M + H]^+^; anal. calcd for C_35_H_40_N_2_O_6_: C 71.90; H 6.90; N 4.79; found: C 71.87; H 6.92, N 4.81.

#### Methyl 2′-(1*H*-indol-5-yl)-12-(3,4,5-trimethoxyphenyl)-13,14-imidazolyl-deisopropyl-dehydroabietate (7j)

White powder solid; mp 189–191 °C; yield: 61.8%; ^1^H NMR (300 MHz, CDCl_3_) *δ*: 1.26 (s, 3H), 1.35 (s, 3H), 1.50–1.80 (m, 7H), 2.02 (m, 1H), 2.43 (d, *J* = 12.1 Hz, 1H), 2.47 (d, *J* = 12.2 Hz, 1H), 3.21 (brs, 2H), 3.70 (s, 3H), 3.93 (s, 3H), 3.96 (s, 6H), 6.62 (s, 1H), 6.82 (s, 2H), 7.25 (s, 1H), 7.31 (s, 1H), 7.46 (d, *J* = 8.5 Hz, 1H), 7.91 (d, *J* = 8.3 Hz, 1H), 8.26 (s, 1H), 8.47 (brs, 1H); ^13^C NMR (75 MHz, CDCl_3_) *δ*: 16.7, 18.8, 21.3, 25.5, 25.6, 36.9, 37.6, 38.7, 45.5, 48.0, 52.1, 56.5, 61.1, 103.2, 106.3, 111.8, 118.4, 119.3, 119.7, 121.1, 122.0, 125.8, 128.2, 129.0, 131.0, 135.2, 137.0, 137.7, 140.9, 144.1, 153.2, 153.6, 179.4; IR (KBr, cm^−1^): 3345, 2959, 2924, 2853, 1724, 1620, 1580, 1498, 1456, 1379, 1258, 1126, 836, 737; MS (ESI): *m*/*z* 593.3 [M + H]^+^; anal. calcd for C_36_H_39_N_2_O_5_: C 72.83; H 6.62; N 7.08; found: C 72.85; H 6.62, N 7.05.

#### Methyl 2′-(1*H*-indol-3-yl)-12-(3,4,5-trimethoxyphenyl)-13,14-imidazolyl-deisopropyl-dehydroabietate (7k)

White powder solid; mp 199–201 °C; yield: 56.2%; ^1^H NMR (500 MHz, CDCl_3_) *δ*: 1.27 (s, 3H), 1.33 (s, 3H), 1.50–1.80 (m, 7H), 1.98 (m, 1H), 2.38 (d, *J* = 12.2 Hz, 1H), 2.46 (d, *J* = 12.0 Hz, 1H), 3.11 (m, 1H), 3.21 (m, 1H), 3.66 (s, 3H), 3.89 (s, 3H), 3.93 (s, 6H), 7.06–7.22 (m, 5H), 7.25 (s, 1H), 7.67 (s, 1H), 8.21 (s, 1H), 9.59 (brs, 1H); ^13^C NMR (75 MHz, CDCl_3_) *δ*: 16.7, 18.8, 21.4, 25.5, 25.7, 36.9, 37.6, 38.7, 45.4, 48.0, 52.1, 56.4, 61.1, 106.1, 106.2, 112.0, 118.2, 120.3, 120.4, 121.3, 123.1, 125.4, 126.1, 127.0, 131.1, 135.3, 136.6, 137.7, 139.9, 144.2, 148.9, 153.6, 179.4; IR (KBr, cm^−1^): 3398, 3058, 2926, 2853, 1725, 1624, 1584, 1498, 1455, 1427, 1377, 1295, 1125, 833, 743; MS (ESI): *m*/*z* 593.3 [M + H]^+^; anal. calcd for C_36_H_39_N_2_O_5_: C 72.83; H 6.62; N 7.08; found: C 72.80; H 6.63, N 7.09.

### Cell lines and culture

The human hepatocarcinoma cell line (SMMC-7721), human breast cancer cell line (MDA-MB-231), human cervical carcinoma cell line (HeLa), mouse colon cancer cell line (CT-26) and human normal hepatocyte cells (QSG-7701) used in this study were all obtained from Keygene Biotech., China. These cell lines were maintained in DMEM medium supplemented with 10% heat-inactivated fetal bovine serum in a humidified atmosphere of 5% CO_2_ at 37 °C.

### Cytotoxic assay

The *in vitro* cytotoxic activities of the benzimidazole derivatives of dehydroabietic acid were evaluated against tested cell lines *via* the MTT colorimetric method. Briefly, different tumor cells were grown in DMEM medium supplemented with 10% fetal bovine serum, penicillin (100 U mL^−1^), and streptomycin (50 μg mL^−1^). Cells were harvested at log phase of growth and seeded in 96-well plates (100 μL per well at a density of 2 × 10^5^ cells mL^−1^). After 24 h incubation at 37 °C and 5% CO_2_ to allow cell attachment, cultures were exposed to various concentrations of the isolated compounds for 48 h. Finally, MTT solution (2.5 mg mL^−1^ in PBS) was added (40 μL per well). Plates were further incubated for 4 h at 37 °C, and the formazan crystals formed were dissolved by adding 150 μL per well of DMSO. Absorption at 570 nm was measured with an ELISA plate reader. The results were expressed as IC_50_ values with standard deviations, which was defined as the concentration at which 50% survival of cells was discerned. Nocodazole was co-assayed as positive control.

### *In vitro* tubulin polymerization inhibition

The tubulin polymerization assay was accomplished using a commercial assay kit (cat. #BK004P) purchased from Cytoskeleton, Inc. (Danvers, MA, USA). Tubulin isolated from porcine brain tissue was used in this tubulin polymerization assay kit. Tubulin polymerizations are followed by an increase in optical density at 340 nm (OD_340_) over a 60 min period at 37 °C due to the light scattered by microtubule polymer. At first, tubulin (4 mg) was disovled in 1 mL of G-PEM buffer containing 80 mM piperazine-*N*,*N*′-bis(2-ethanesulfonic acid) sequisodium salt (PIPES, pH 6.9), 2.0 mM MgCl_2_, 0.5 mM EGTA and 1 mM GTP to prepare tubulin solution. Subsequently, 10 μL of 10 × stock solutions of the tested compounds at different concentrations were added to a prewarmed 96-well plate and incubated at 37 °C for 2 min, then 90 μL of tubulin solution was also added into each well to initiate the reaction. The optical intensity (OD_340_) of each well was recorded every 60 s for 60 min (Cytation 3 Cell Imaging Multi-Mode Reader, BioTek Instruments, Inc., USA). Colchicine and taxol were co-assayed as the positive control and negative control, respectively. G-PEM buffer was used as the blank control.

### Immunofluorescence

The intracellular microtubule morphology was investigated using the protocol previously reported.^52^ For the immunofluorescence studies, SMMC-7721 cells (3 × 10^5^ cells per well) were seeded on 10 mm^2^ confocal culture dishes (NEST Biotechnology, China) for 24 h and then incubated in the absence or presence of compound 6j at the indicated concentrations for another 12 h. After being washed with phosphate buffer solution (PBS) and fixed in 4% paraformaldehyde for 15 min, the cells were permeabilized with 0.5% Triton X-100 for 15 min and blocked for 30 min with 10% goat serum. Then, the cells were incubated with mouse anti-tubulin antibody (ZSGB-Bio, China) at 4 °C overnight and incubated with goat anti-mouse IgG/Alexa-Fluor 488 antibody (ZSGB-Bio, China) at room temperature for 1 h. After the nuclei were stained with Hoechst 33258 (Beyotime Biotech., China) in the dark at room temperature for 30 min, the samples were immediately visualized on a Zeiss LSM 570 laser scanning confocal microscope (Carl Zeiss, Germany).

### Wound healing assay

SMMC-7721 cells (5 × 10^5^ cells per well) were grown in a 12-well plate for 24 h. Scratches were made in confluent monolayers using 200 μL pipette tip. Then, wounds were washed twice with PBS to remove non-adherent cell debris. The media containing different concentrations (0, 0.1, 0.2 and 0.5 μM) of compound 6j were added to the corresponding wells. Cells which migrated across the wound area were photographed under the phase contrast microscope (Olympus 1X71 Inverted System Microscope, Olympus, Japan) after 24 and 48 h treatment.

### Cell cycle analysis

For the flow cytometric analysis of the DNA content, SMMC-7721 cells were seeded in 6-well plates (5 × 10^5^ cells per well) and incubated in the absence or presence of compound 6j at indicated concentrations for 24 h. After treatment, cells were detached with 0.25% trypsin, harvested by centrifugation, washed twice with ice-cold PBS and then fixed and permeabilized with ice-cold ethanol at 4 °C overnight. Ethanol was removed by centrifugation, and the cells were washed twice and resuspended in ice-cold PBS. After this, the cells were treated with 100 μL of 100 μg mL^−1^ RNAse A at 37 °C for 30 min, followed by incubation with 400 μL of 1 mg mL^−1^ DNA staining solution propidium iodide (PI) in the dark at 4 °C for 30 min. The samples were then analyzed for their DNA content by flow cytometry (Becton-Dickinson FACSCalibur, New York, USA).

### Annexin V-FITC/PI dual staining assay

Apoptosis was discriminated with the Annexin V-FITC/PI dual staining assay. SMMC-7721 cells were seeded at 1 × 10^5^ cells per well in 10% fetal calf serum (FBS)-DMEM into six-well plates and treated with or without compound 6j for 24 h. The cells were detached with 0.25% trypsin, washed twice with ice-cold PBS and then resuspended in 500 μL of 1× binding buffer (0.1 M Hepes/NaOH (pH 7.4), 1.4 M NaCl, 25 mM CaCl_2_). The solution was transferred into 5 mL culture tubes, and 5 μL of Annexin V-FITC (BD, Pharmingen) and 5 μL of PI were added to each tube. The cells were gently vortexed, and incubated for 30 min at 25 °C in the dark. The analysis was performed on a flow cytometry (Becton-Dickinson FACSCalibur, New York, USA).

### ROS generation assay

ROS generation assay was performed by using the reactive oxygen species assay kit (Beyotime Biotech., China). Intracellular ROS generation was tested through dichlorodihydro fluorescein diacetate (DCFH-DA) assay. DCFH-DA is taken up by SMMC-7721 cells, and then activated by esterase-mediated cleavage of acetate to form dichlorodihydro fluorescein (DCFH), which is trapped in the cells. DCFH is converted to fluorescein DCF in the presence of ROS. SMMC-7721 cells were seeded in six-well plates and incubated with different concentrations of compound 6j for 24 h. After removing the compound solution, cells were treated with 10 μM of DCFH-DA at 37 °C for 20 min. Subsequently, the cells were washed with PBS for three times and then exposed to light. Immediately after light exposure, cell images were acquired through an inverted fluorescence microscope (Olympus 1X71 Inverted System Microscope, Olympus, Japan).

### JC-1 mitochondrial membrane potential assay

The JC-1 mitochondrial membrane potential assay kit (Keygene Biotech., China) was employed to measure mitochondrial depolarization in SMMC-7721 cells. Briefly, cells cultured in six-well plates after indicated treatments were incubated with an equal volume of JC-1 staining solution (5 μg mL^−1^) at 37 °C for 20 min and rinsed twice with PBS. Mitochondrial membrane potentials were monitored by determining the relative amounts of dual emissions from mitochondrial JC-1 monomers or aggregates using flow cytometry (Becton-Dickinson FACSCalibur, New York, USA). Mitochondrial depolarization is indicated by an increase in the percentage of cells with low Δ*Ψ*_m_ (green fluorescence, lower right quadrant) compared with cells with high Δ*Ψ*_m_ (red fluorescence, upper right quadrant).

### Molecular modelling

X-ray structures of the tubulin co-crystal was retrieved from the PDB (accession code: 1SA0)^53^ and prepared using the Protein Preparation Wizard workflow from Schrödinger suite,^50^ including optimization of the hydrogen bond network and a short energy minimization with position restraints on heavy atoms using the OPLS_2005 force field. Ligands were then freely docked in the colchicine binding site located between chains A and B using the standard protocol implemented in Maestro, version 10.1. van der Waals (vdW) scaling of 0.8 and partial cut-off of 0.15 were set to soften the potential for non-polar sites, and no constraints were specified. The best docked pose ranked by GlideScore value was recorded, and saved for each ligand. The structures of complexes were analysed for interactions and the 3D pose of the most active compound 6j was displayed using Discovery studio 3.5 client.

## Conflicts of interest

There are no conflicts to declare.

## Supplementary Material

RA-008-C8RA02078G-s001
