# Supplementary material for: Synthesis and biological evaluation of 2-aryl-benzimidazole derivatives of dehydroabietic acid as novel tubulin polymerization inhibitors
Source: RSC Adv. 2018 May 16;8(31):17511–26. doi: 10.1039/c8ra02078g (PMC9080489; doi:10.1039/c8ra02078g)
Supplement: RA-008-C8RA02078G-s001 [file RA-008-C8RA02078G-s001.pdf]

Electronic Supplementary Material (ESI) for RSC Advances.
This journal is © The Royal Society of Chemistry 2018

Electronic Supplementary Information

Synthesis and biological evaluation of 2-aryl-benzimidazole derivatives of dehydroabietic acid as novel tubulin polymerization inhibitors

Ting-Ting Miao,[1] Xu-Bing Tao,[1] Dong-Dong Li, Hao Chen, Xiao-Yan Jin, Yi Geng, Shi-Fa Wang and Wen Gu *

Jiangsu Provincial Key Lab for the Chemistry and Utilization of Agro-forest Biomass, Jiangsu Key Lab of Biomass-based Green Fuels and Chemicals, College of Chemical Engineering, Nanjing Forestry University, Nanjing 210037, P. R. China

* Corresponding author.
E-mail address: njguwen@163.com

[1] These two authors contributed equally to this study.

Table of Content:

Fig. S1 ~ Fig. S22 ^{1}H and ^{13}C NMR spectra of compounds **6a-k**.

Fig. S23 ~ Fig. S44 ^{1}H and ^{13}C NMR spectra of compounds **7a-k**.

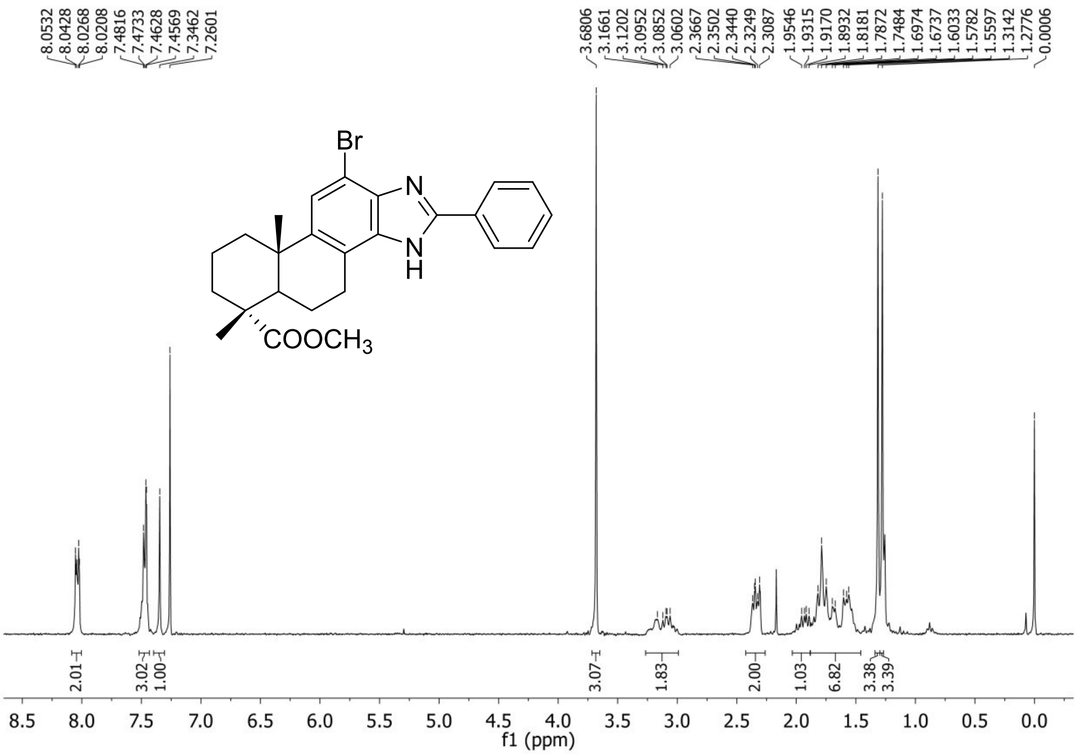

Fig. S1 ^{1}H NMR spectrum of compound **6a** (300 MHz, $CDCl_3$)

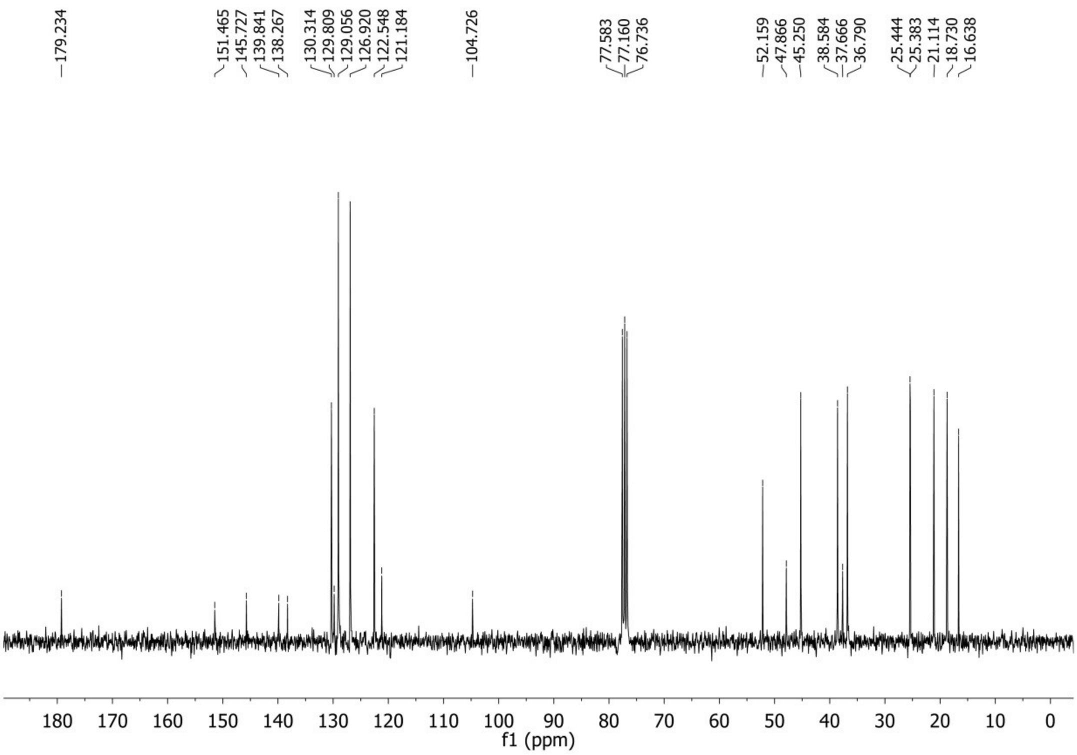

Fig. S2 ^{13}C NMR spectrum of compound **6a** (75 MHz, $CDCl_3$)

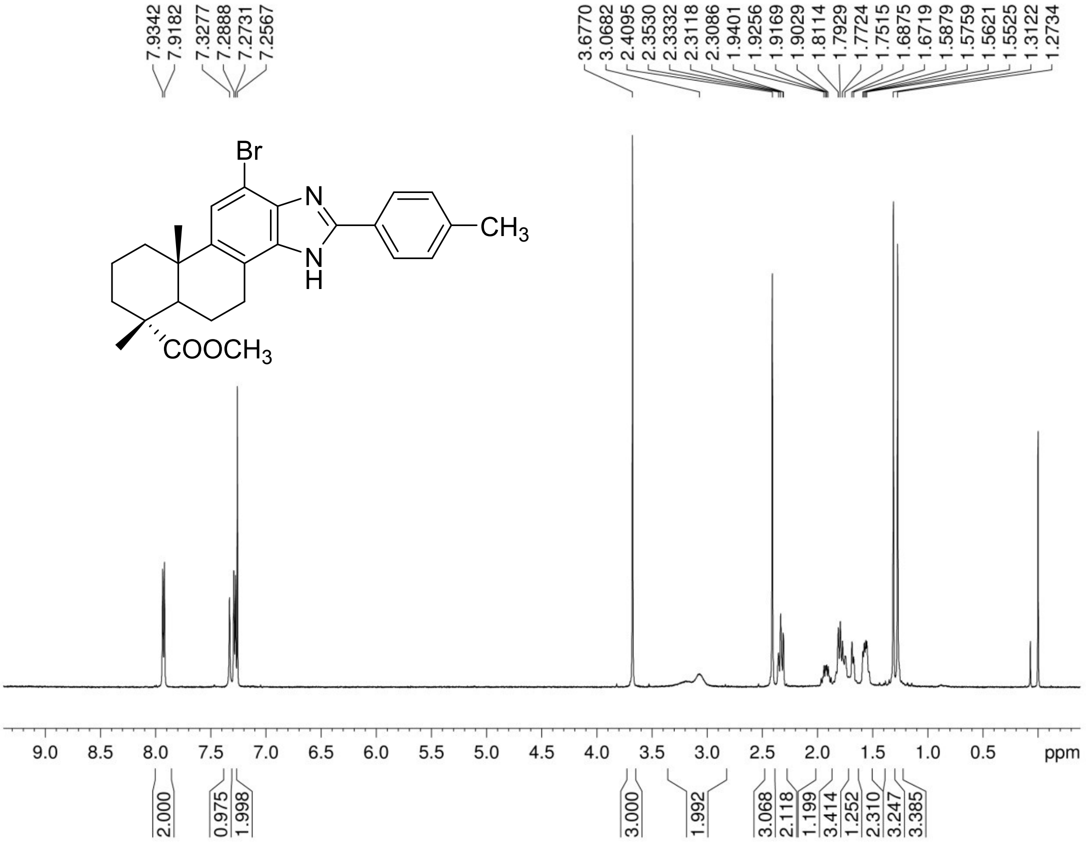

Fig. S3 ^{1}H NMR spectrum of compound **6b** (500 MHz, $CDCl_3$)

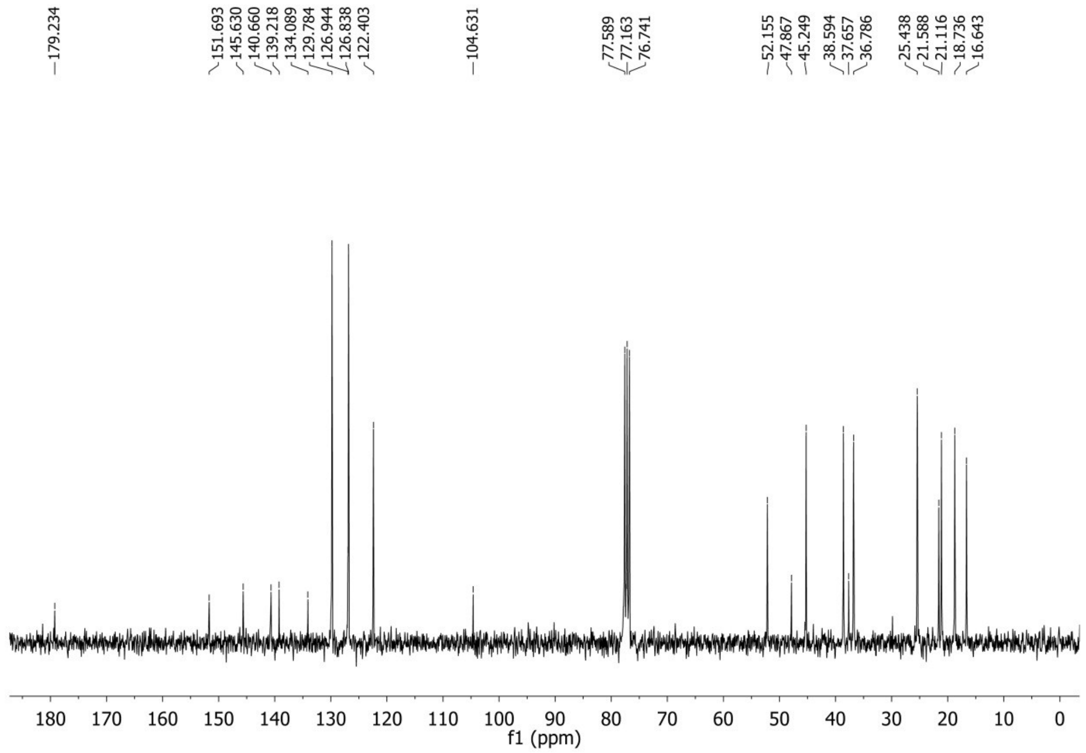

Fig. S4 ^{13}C NMR spectrum of compound **6b** (75 MHz, $CDCl_3$)

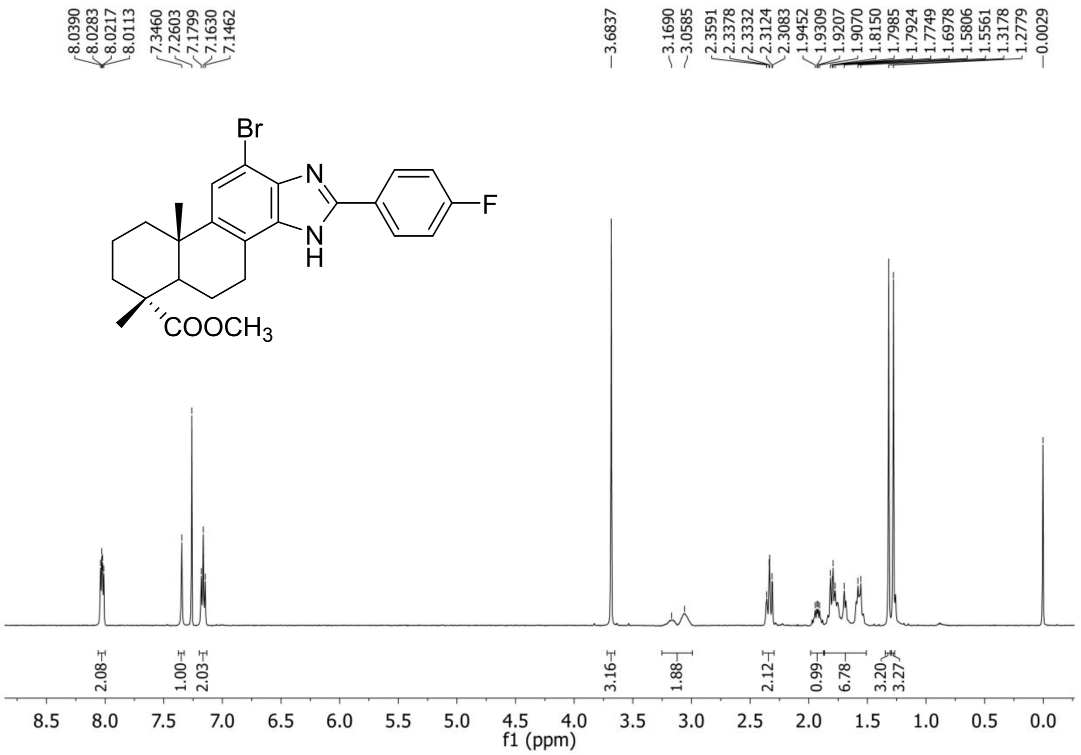

Fig. S5 ^{1}H NMR spectrum of compound **6c** (500 MHz, $CDCl_3$)

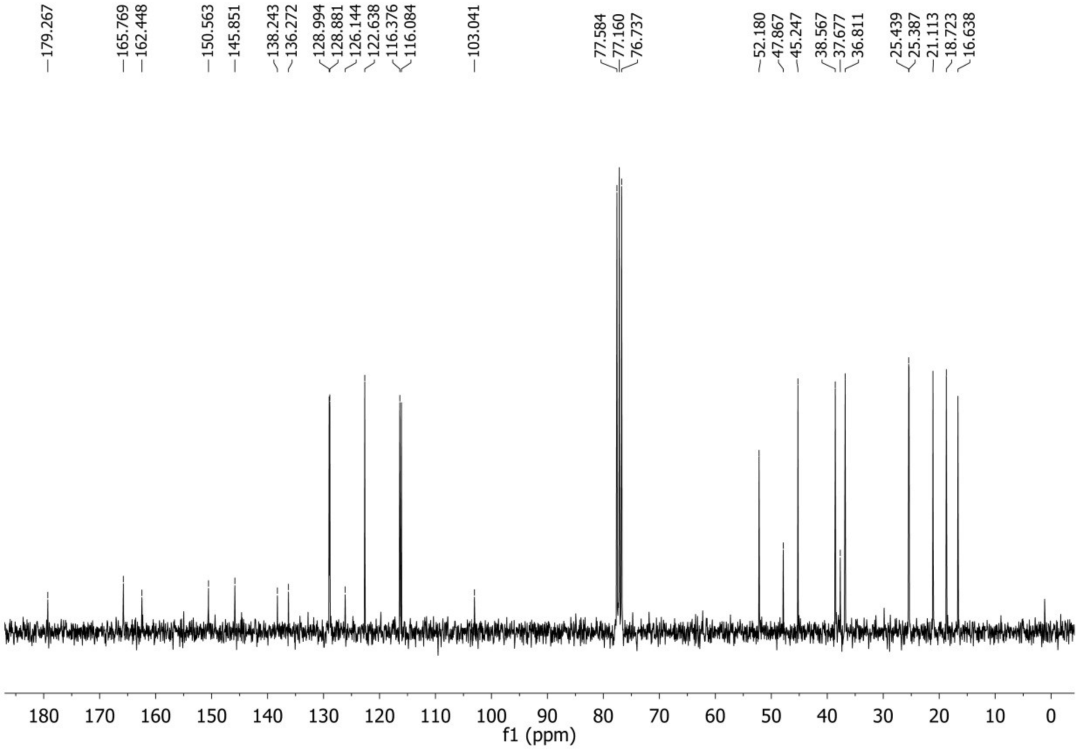

Fig. S6 ^{13}C NMR spectrum of compound **6c** (75 MHz, $CDCl_3$)

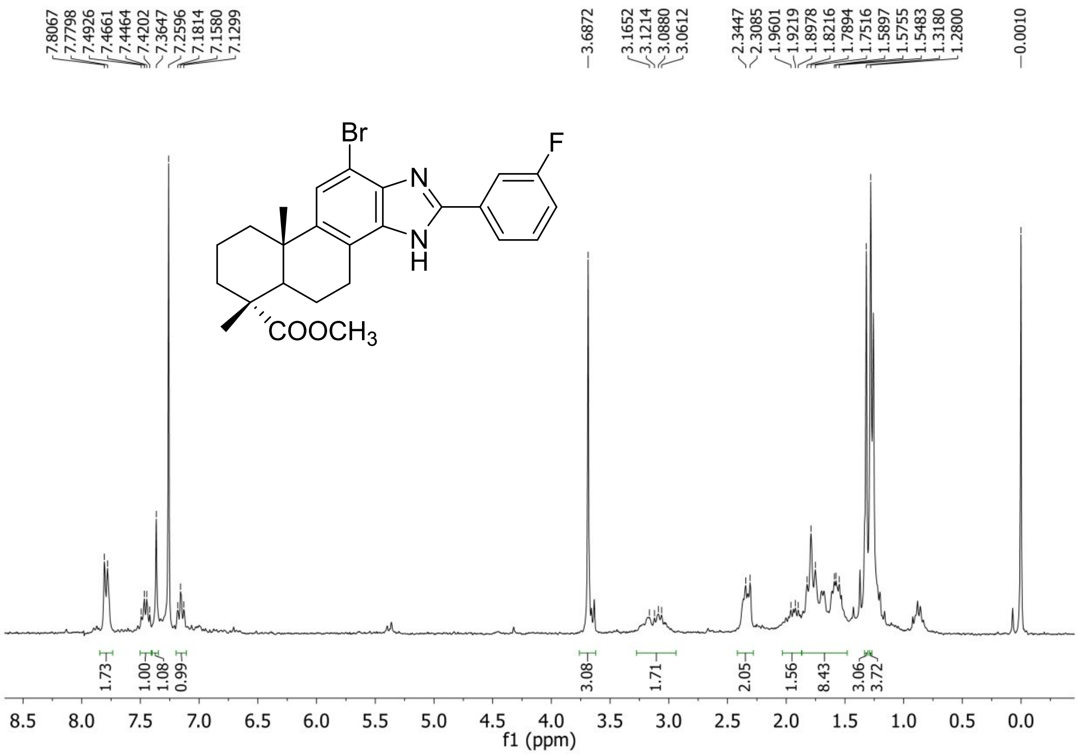

Fig. S7 ^{1}H NMR spectrum of compound **6d** (300 MHz, $CDCl_3$)

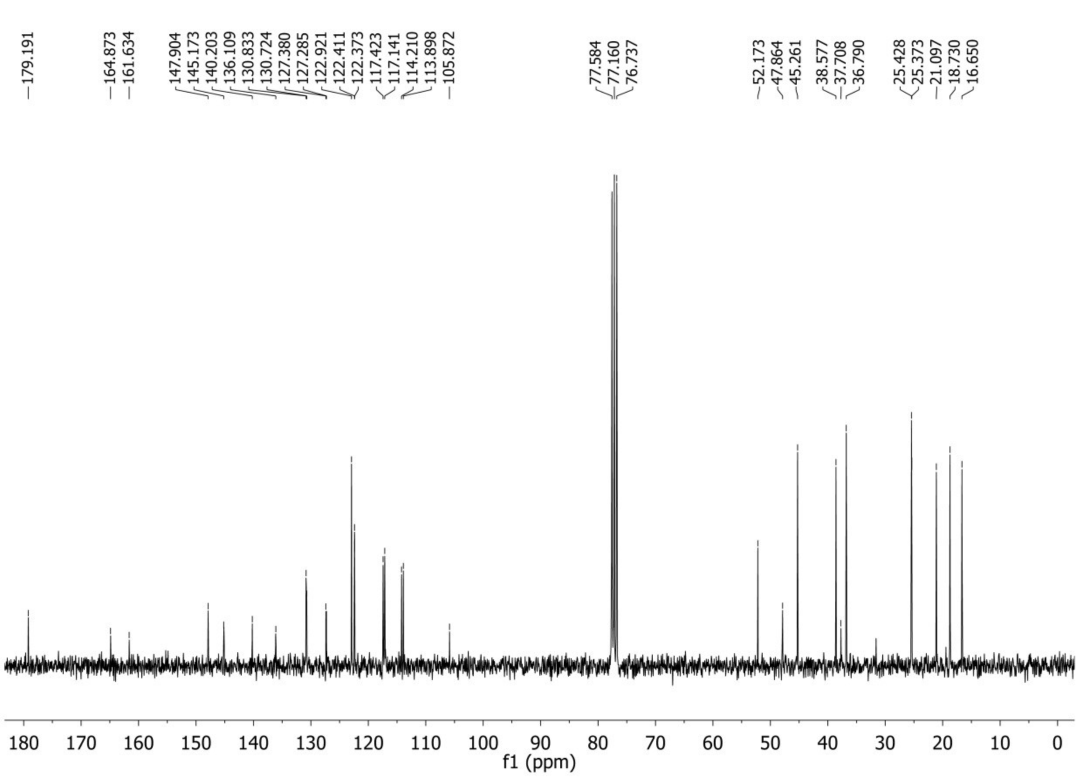

Fig. S8 ^{13}C NMR spectrum of compound **6d** (75 MHz, $CDCl_3$)

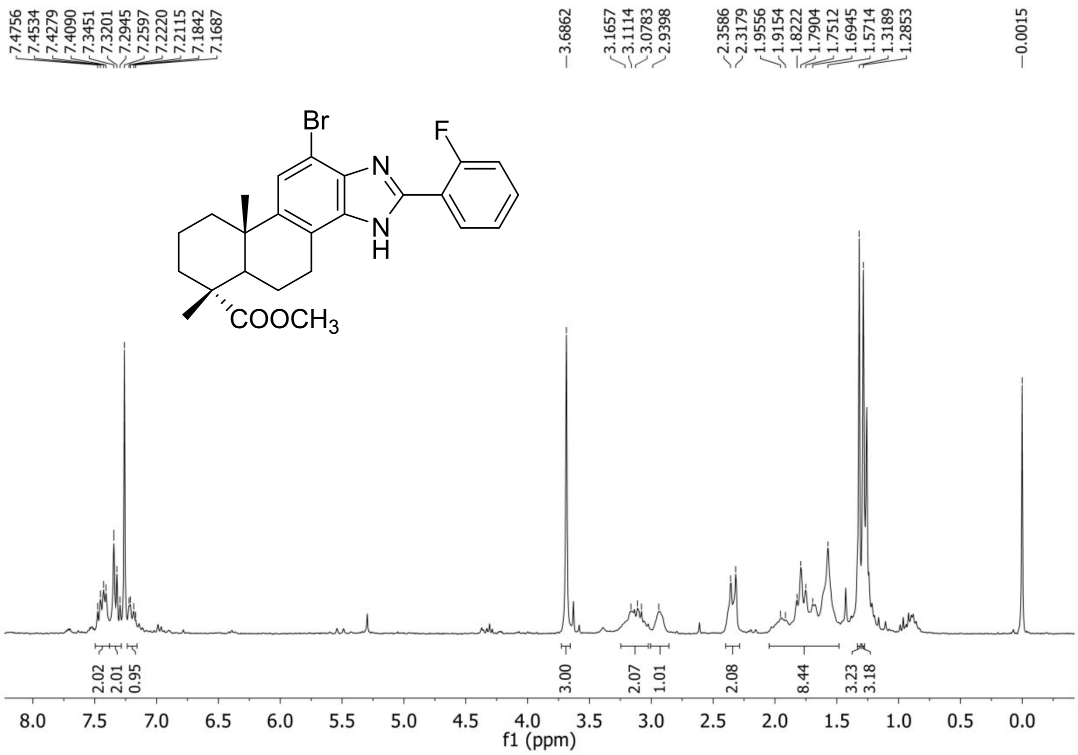

Fig. S9 ^{1}H NMR spectrum of compound **6e** (300 MHz, $CDCl_3$)

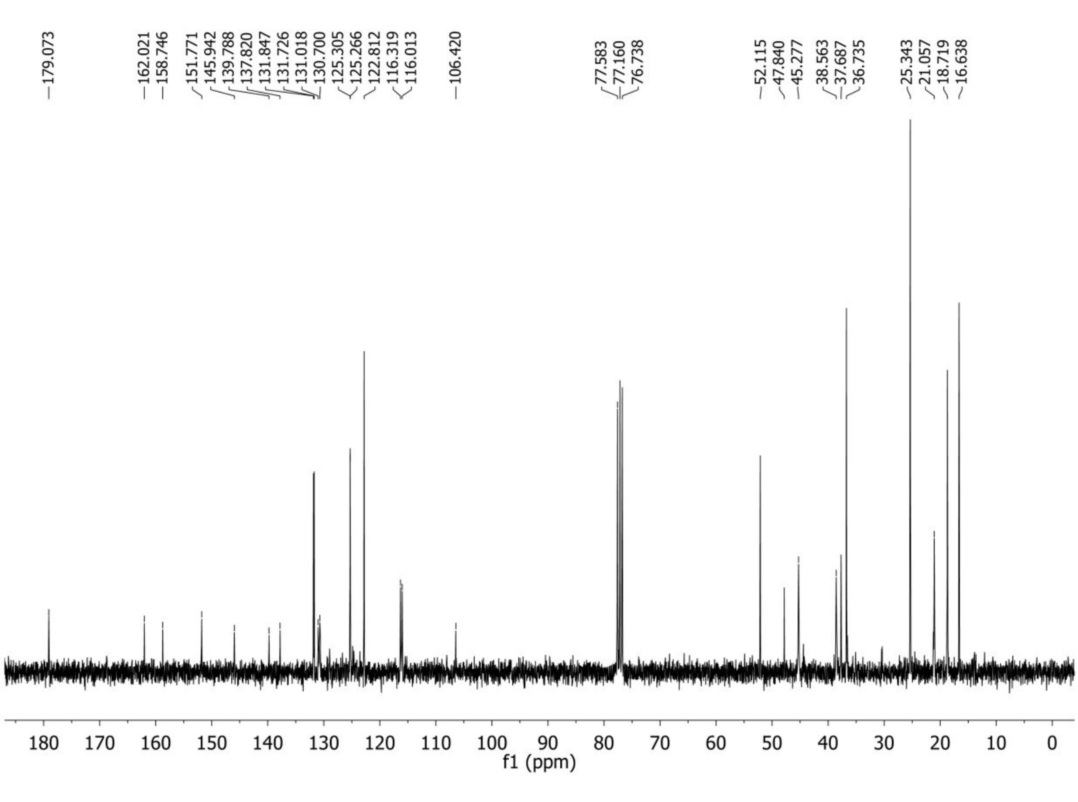

Fig. S10 ^{13}C NMR spectrum of compound **6e** (75 MHz, $CDCl_3$)

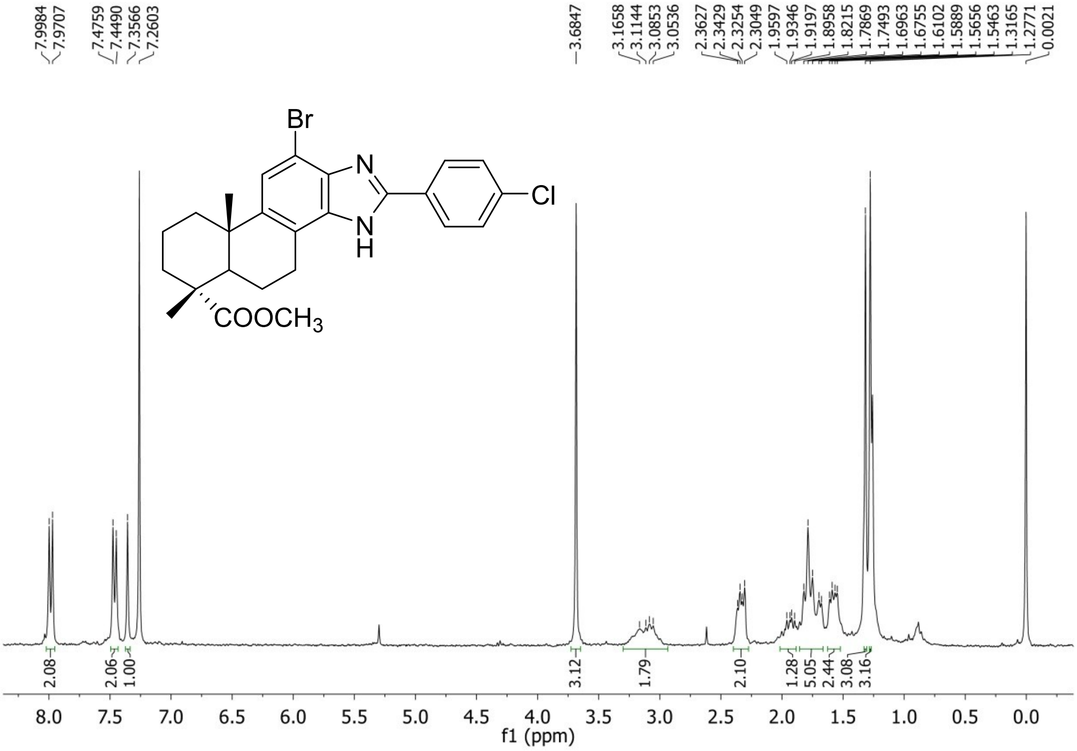

Fig. S11 ^{1}H NMR spectrum of compound **6f** (300 MHz, $CDCl_3$)

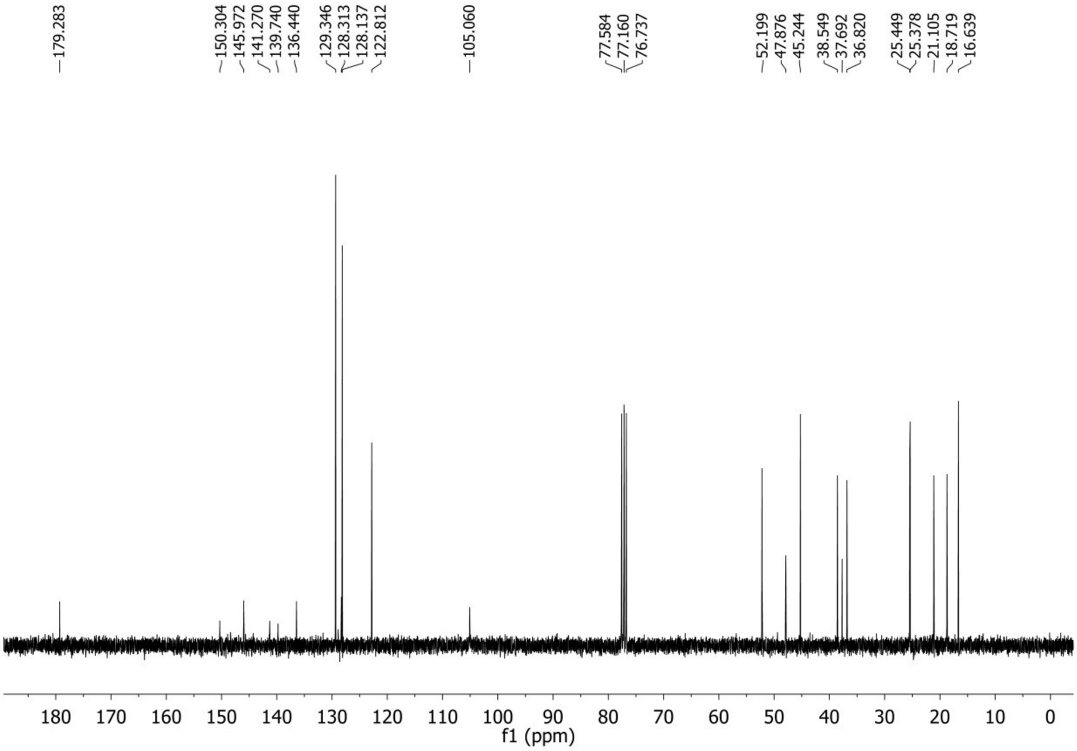

Fig. S12 ^{13}C NMR spectrum of compound **6f** (75 MHz, $CDCl_3$)

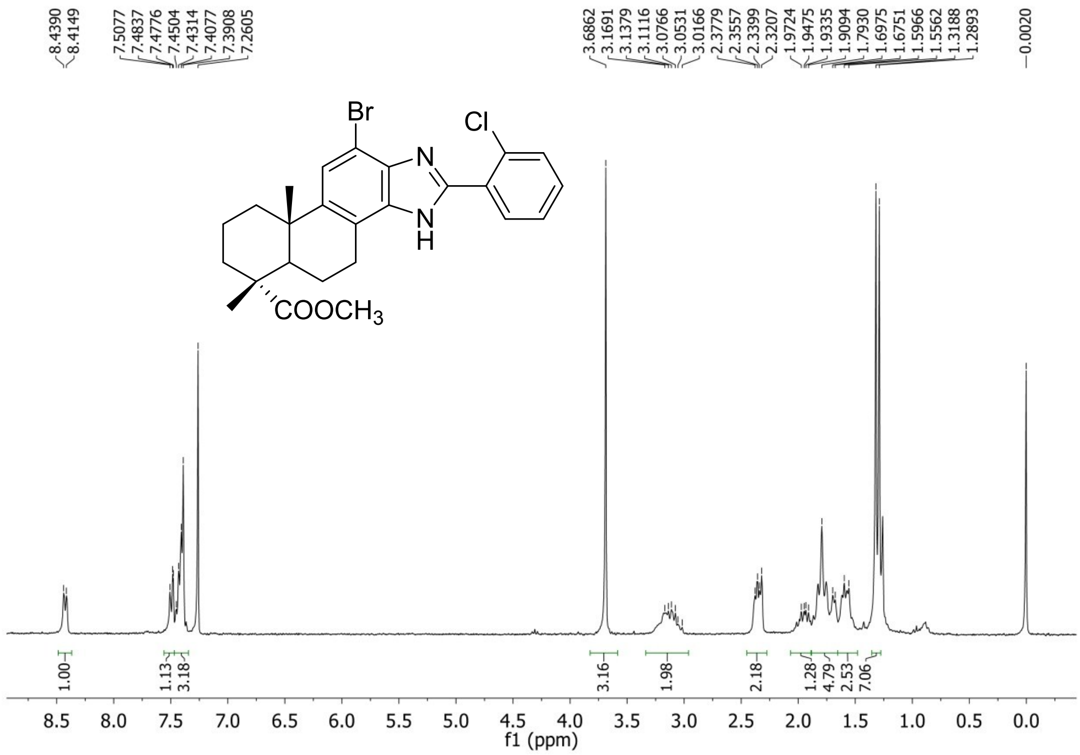

Fig. S13 ^{1}H NMR spectrum of compound **6g** (300 MHz, $CDCl_3$)

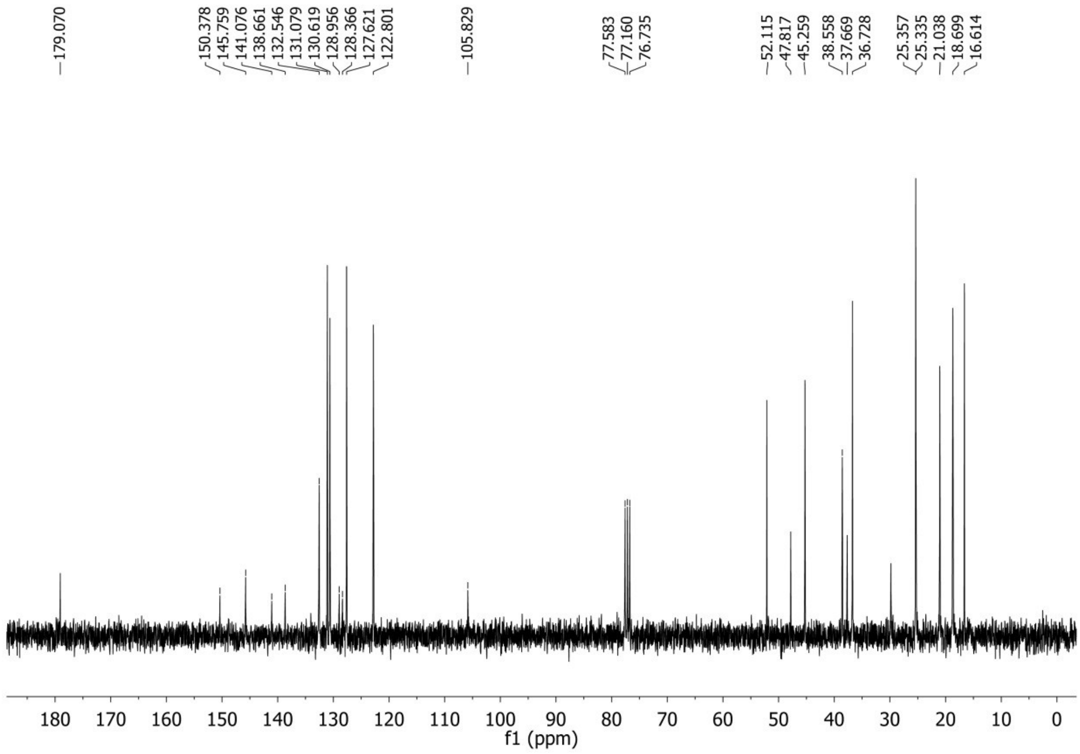

Fig. S14 ^{13}C NMR spectrum of compound **6g** (75 MHz, $CDCl_3$)

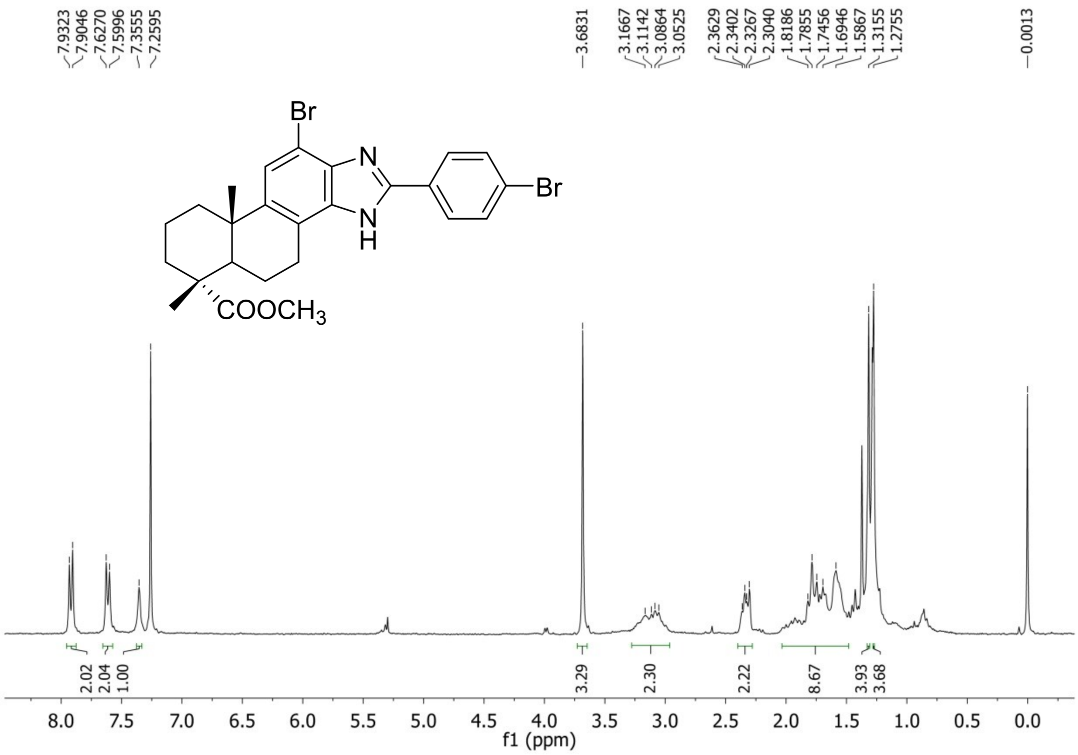

Fig. S15 ^{1}H NMR spectrum of compound **6h** (300 MHz, $CDCl_3$)

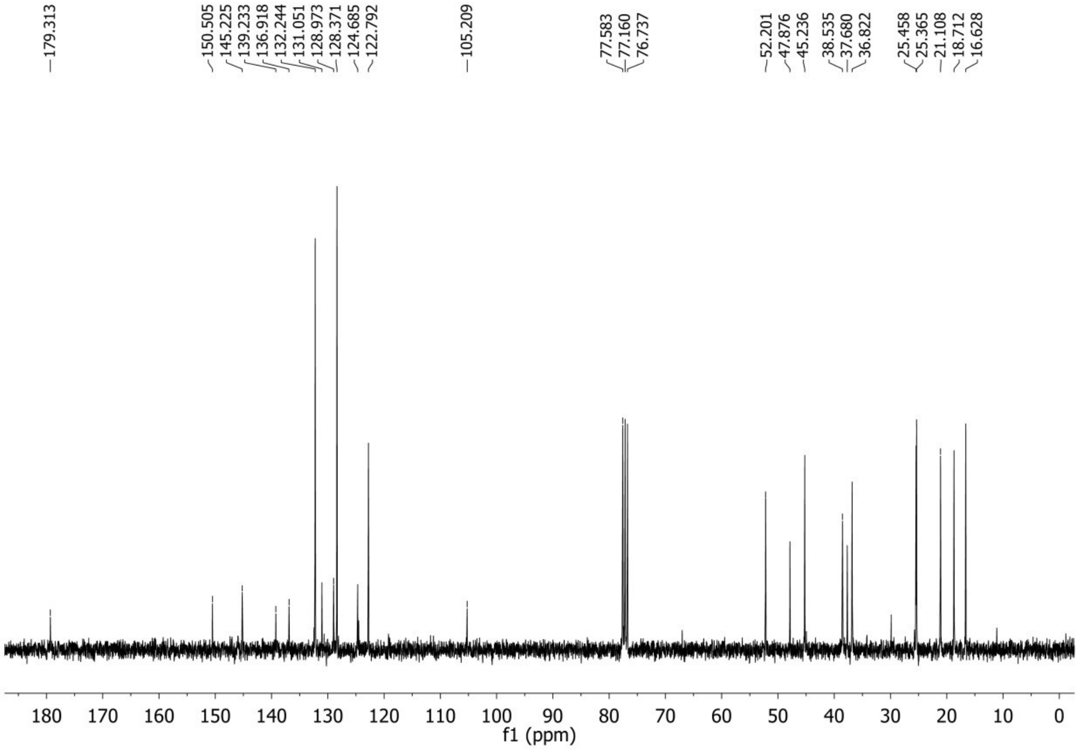

Fig. S16 ^{13}C NMR spectrum of compound **6h** (75 MHz, $CDCl_3$)

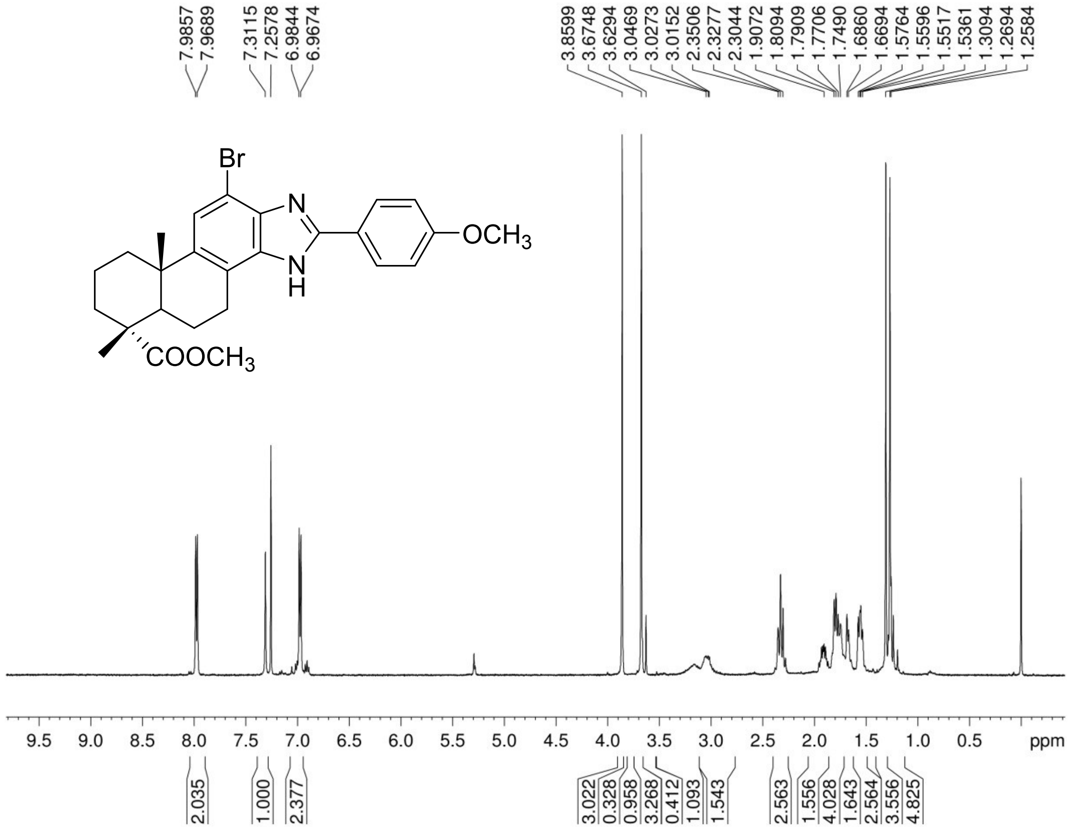

Fig. S17 ^{1}H NMR spectrum of compound **6i** (500 MHz, $CDCl_3$)

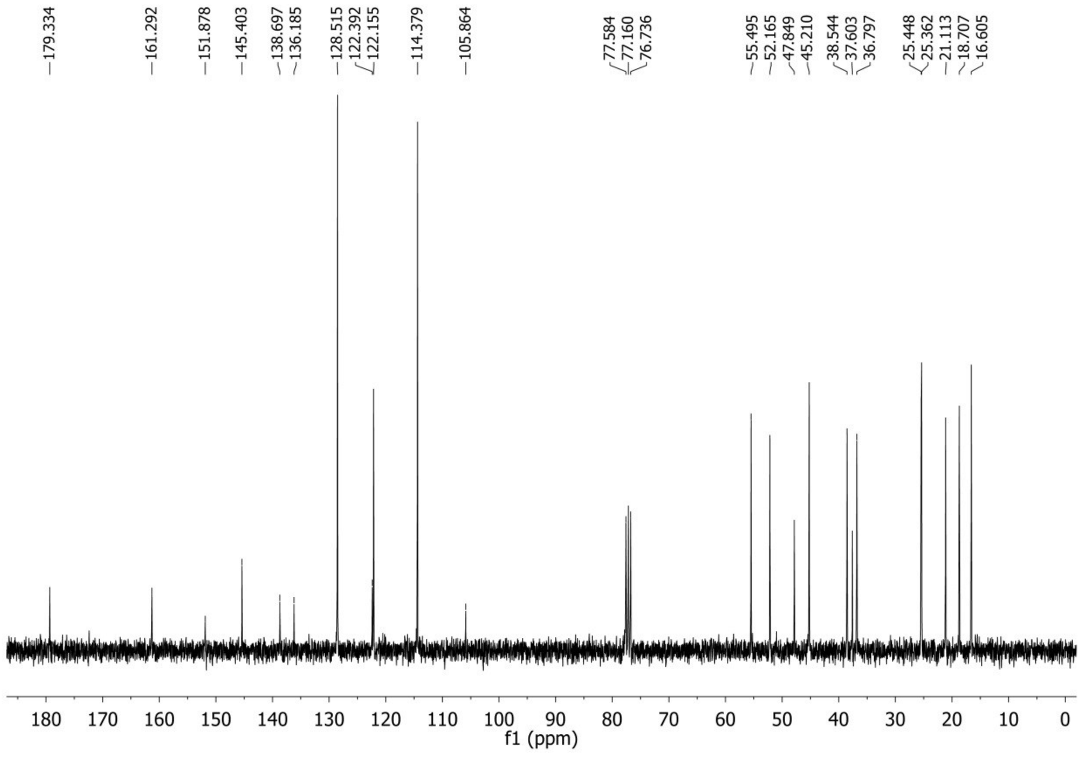

Fig. S18 ^{13}C NMR spectrum of compound **6i** (75 MHz, $CDCl_3$)

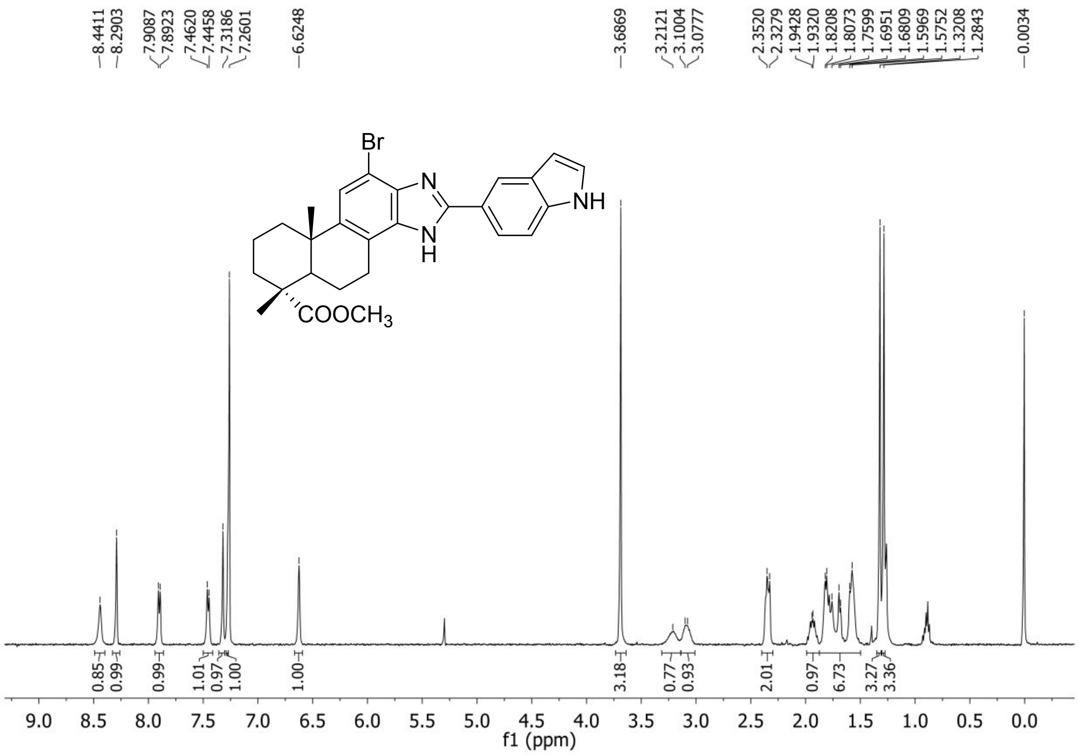

Fig. S19 ^{1}H NMR spectrum of compound **6j** (500 MHz, $CDCl_3$)

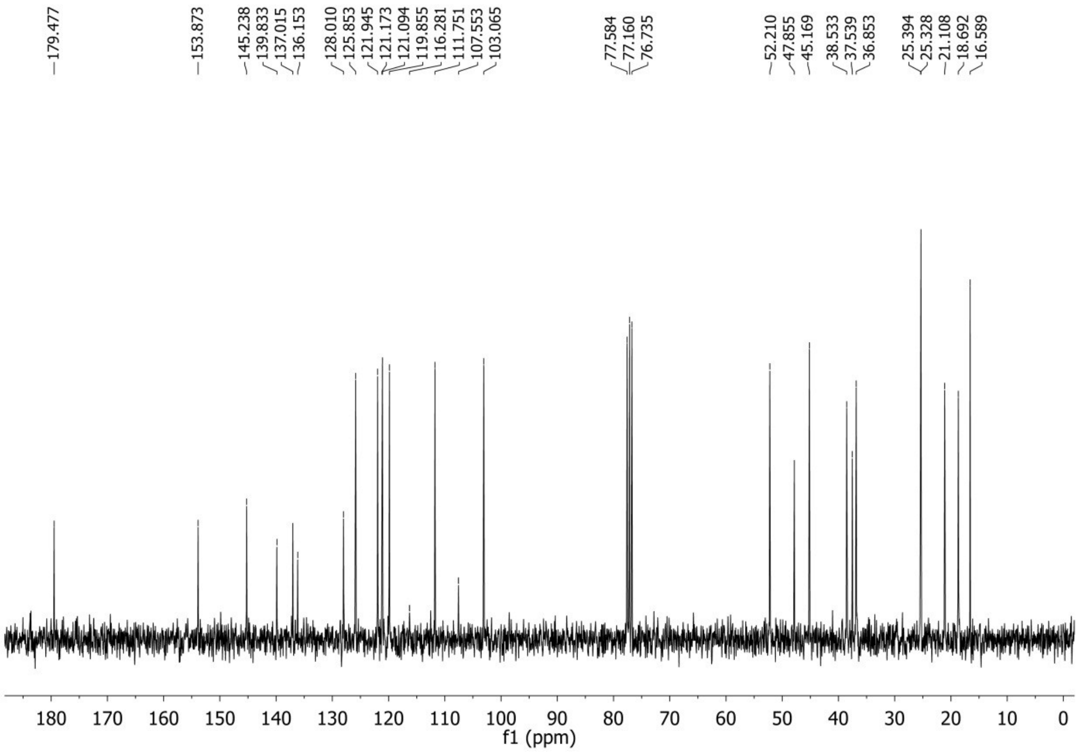

Fig. S20 ^{13}C NMR spectrum of compound **6j** (75 MHz, $CDCl_3$)

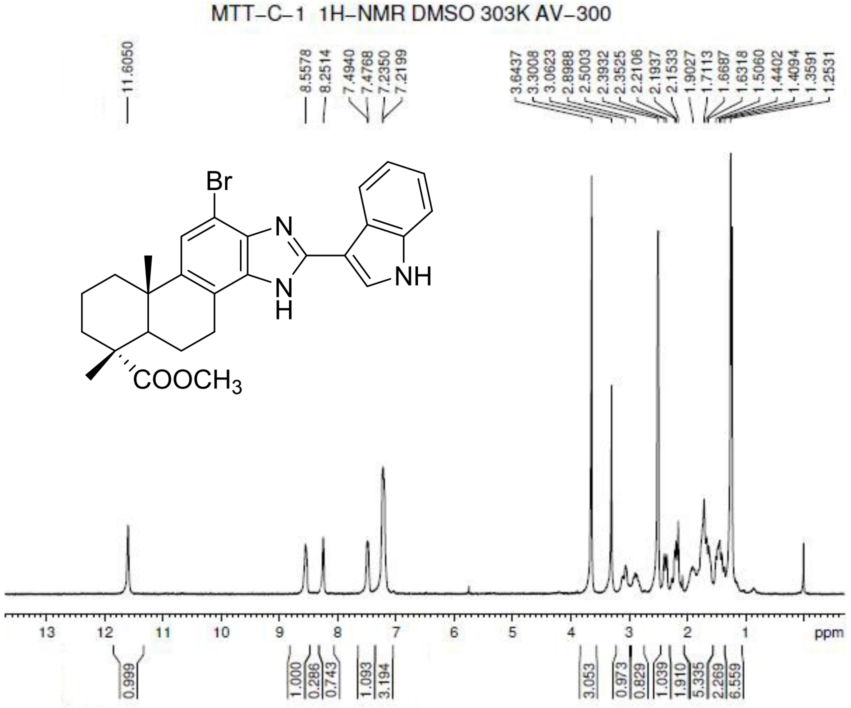

Fig. S21 ^{1}H NMR spectrum of compound **6k** (300 MHz, DMSO-d_6)

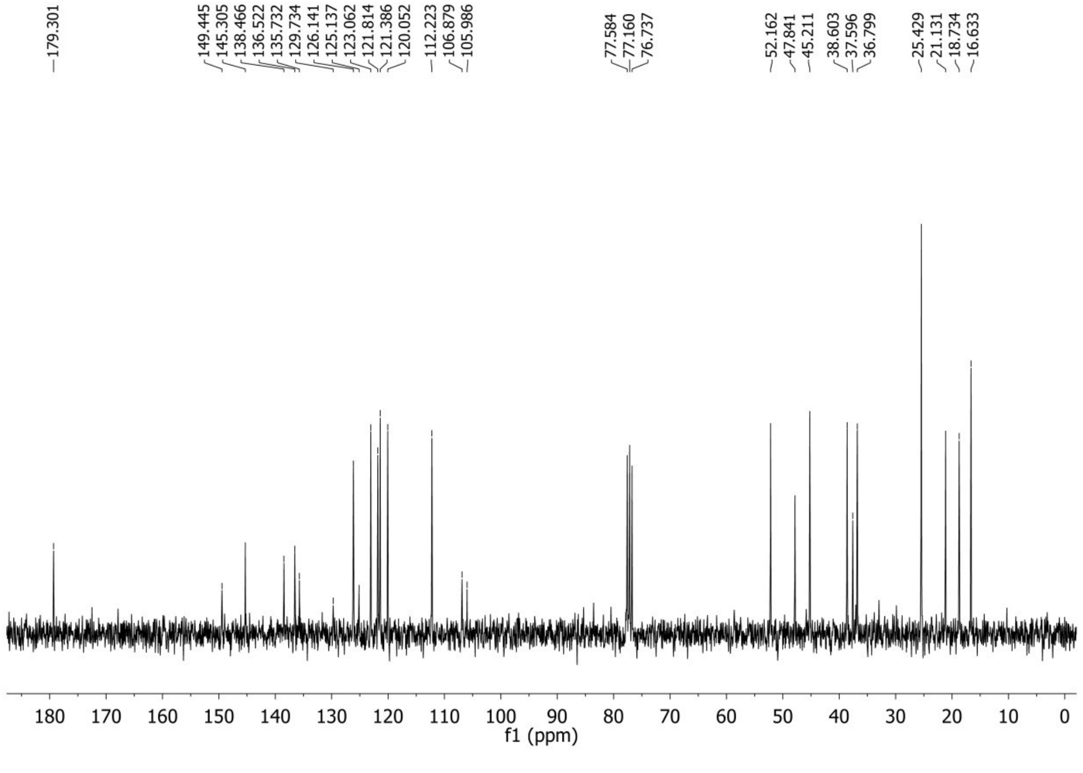

Fig. S22 ^{13}C NMR spectrum of compound **6k** (75 MHz, $CDCl_3$)

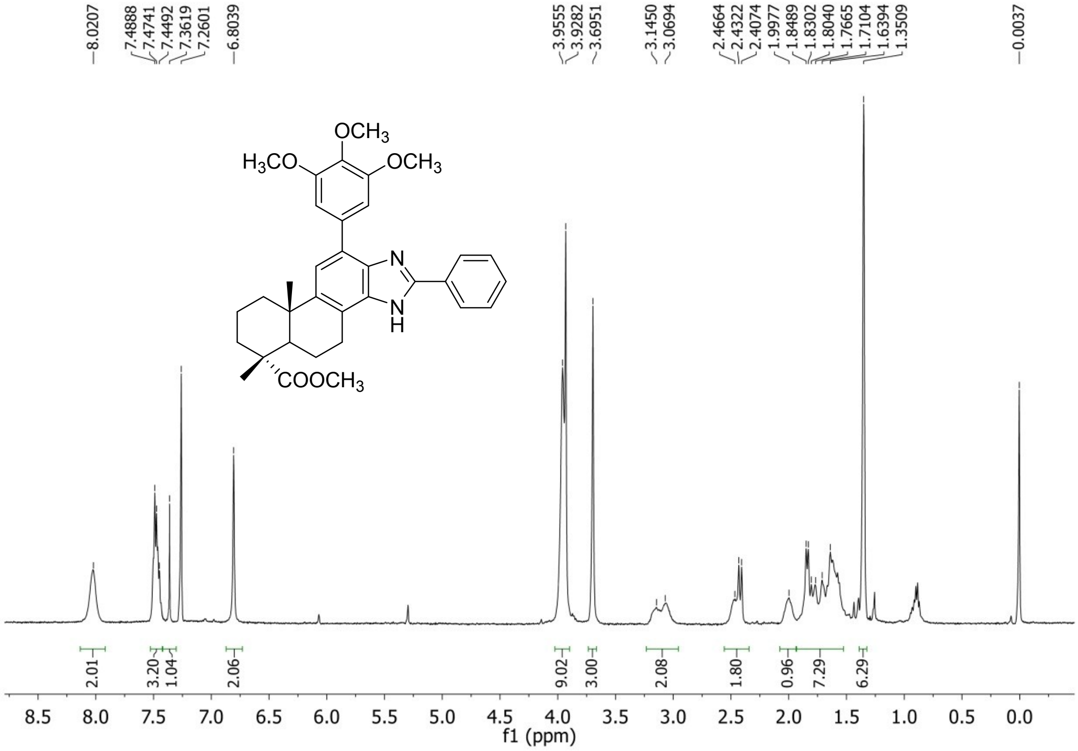

Fig. S23 ^{1}H NMR spectrum of compound **7a** (500 MHz, $CDCl_3$)

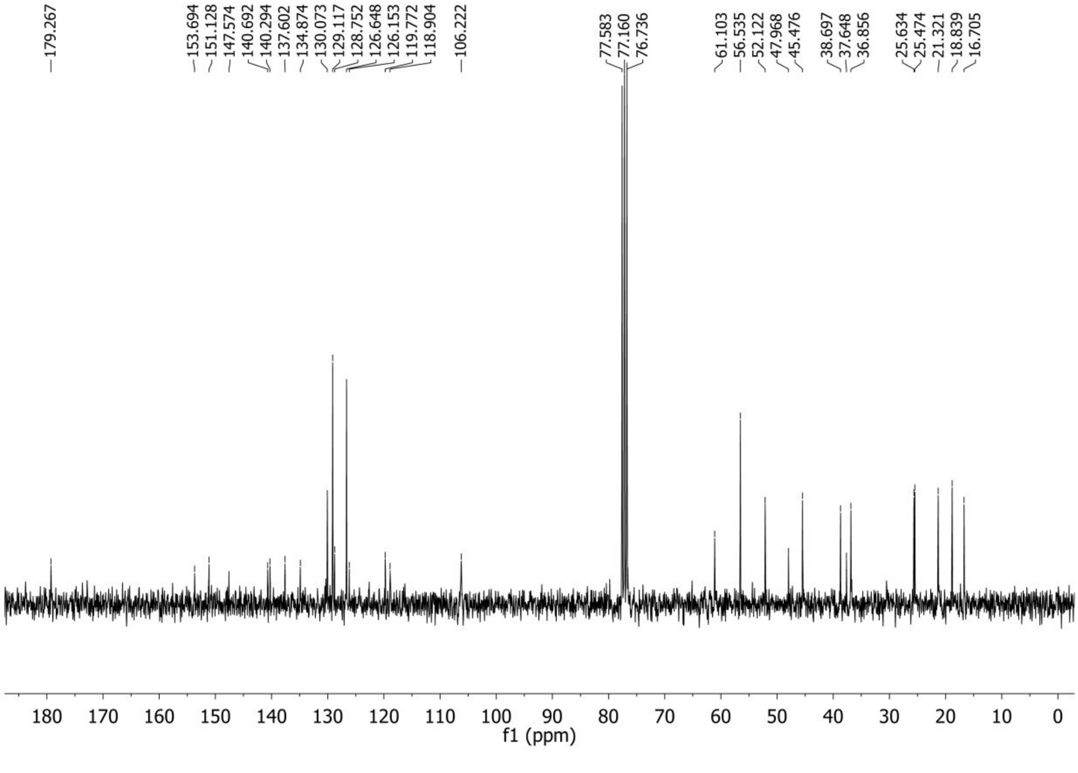

Fig. S24 ^{13}C NMR spectrum of compound **7a** (75 MHz, $CDCl_3$)

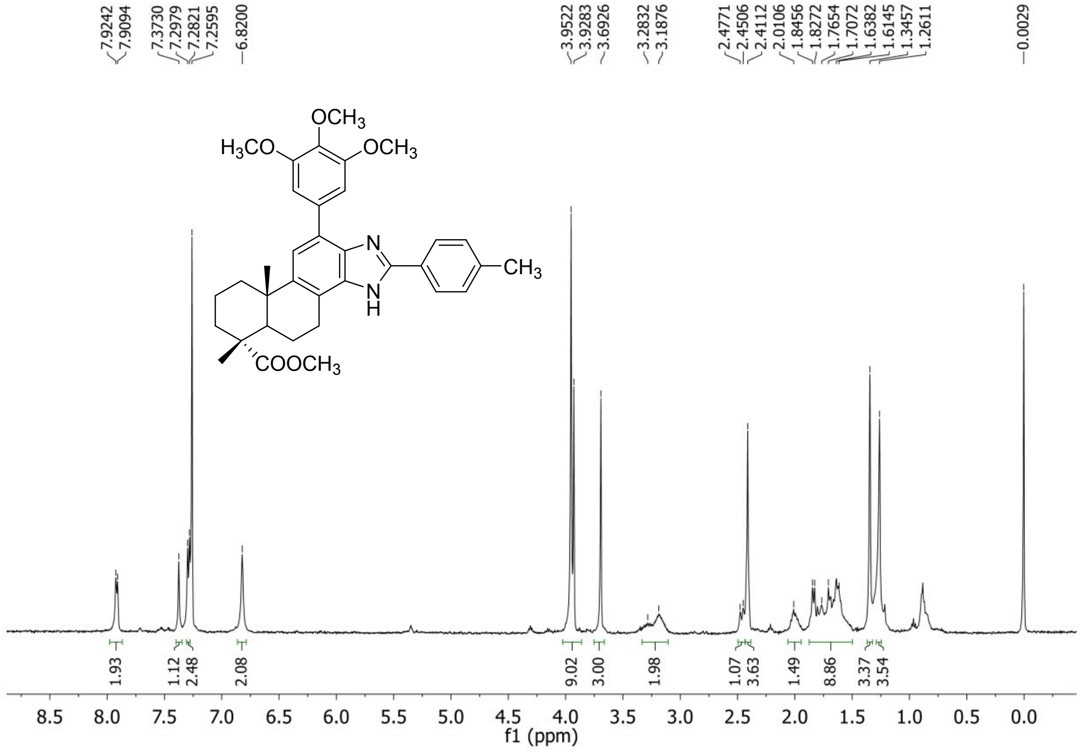

Fig. S25 ^{1}H NMR spectrum of compound **7b** (500 MHz, $CDCl_3$)

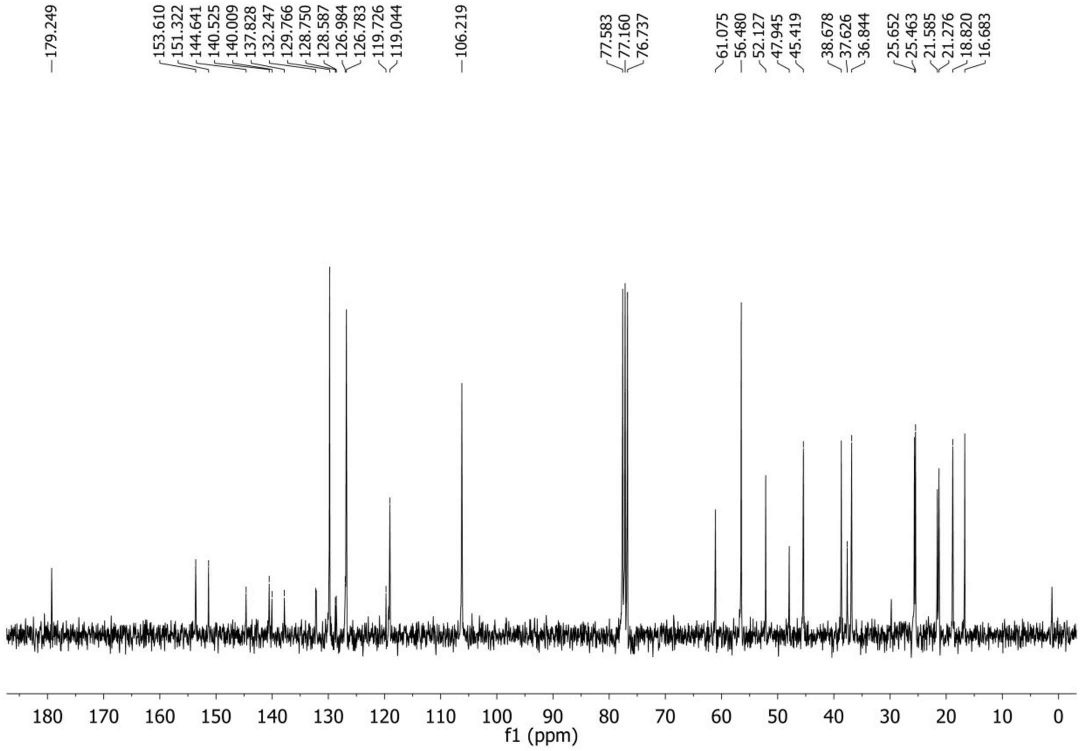

Fig. S26 ^{13}C NMR spectrum of compound **7b** (75 MHz, $CDCl_3$)

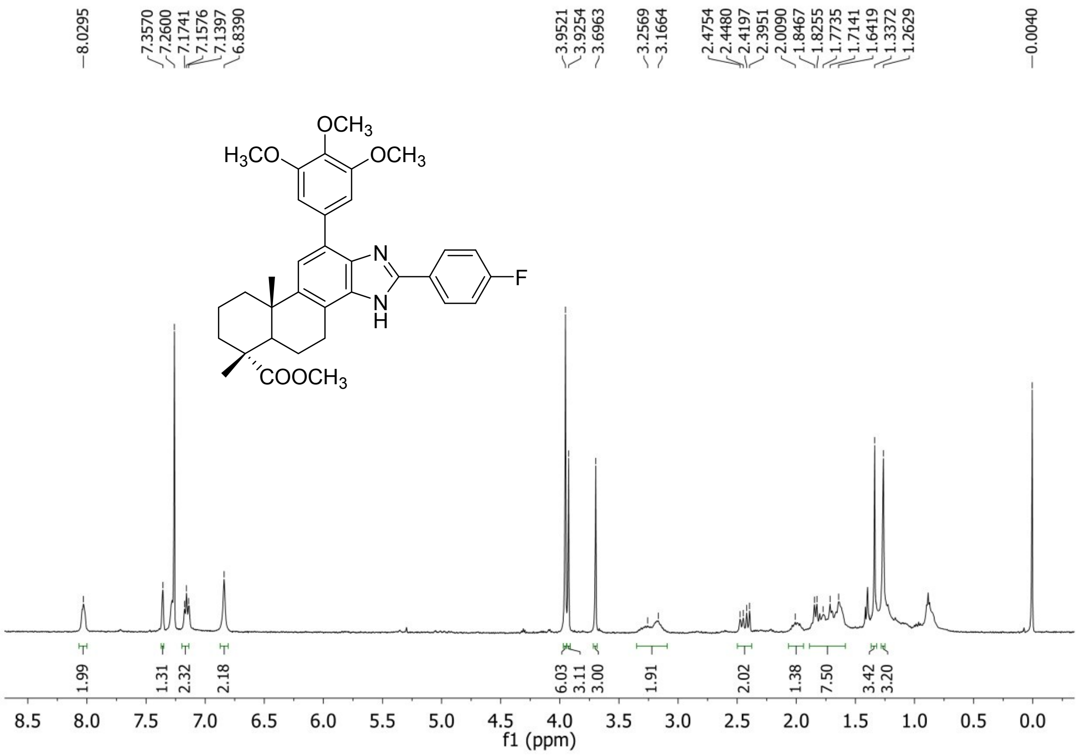

Fig. S27 ^{1}H NMR spectrum of compound **7c** (500 MHz, $CDCl_3$)

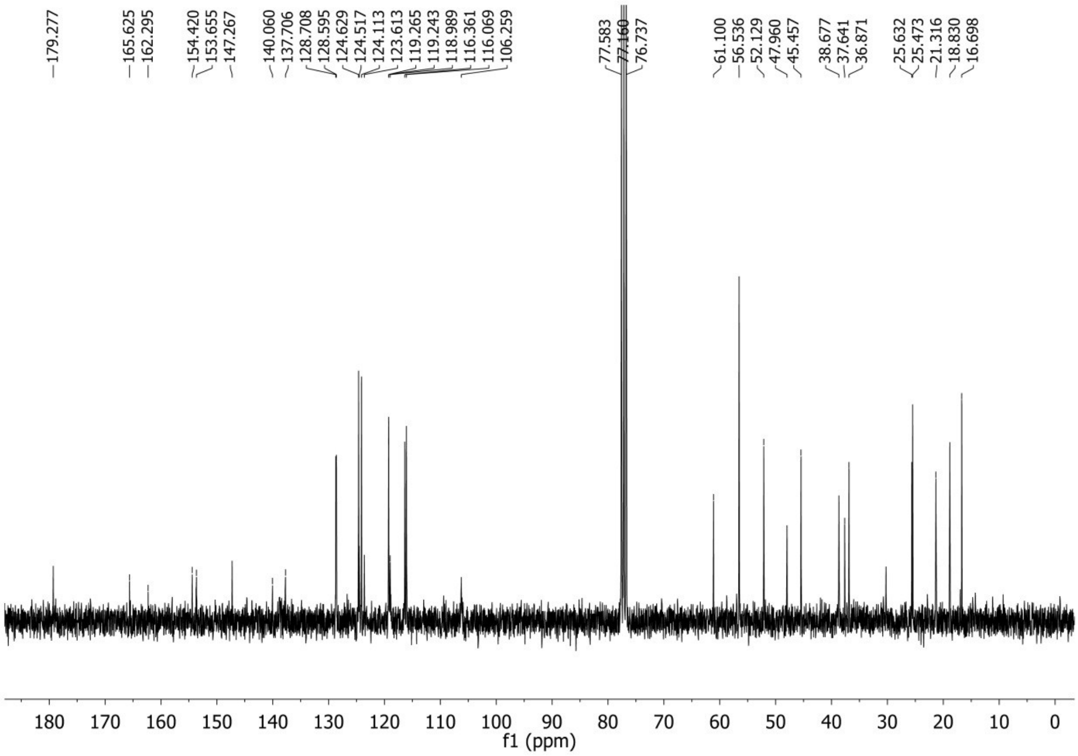

Fig. S28 ^{13}C NMR spectrum of compound **7c** (75 MHz, $CDCl_3$)

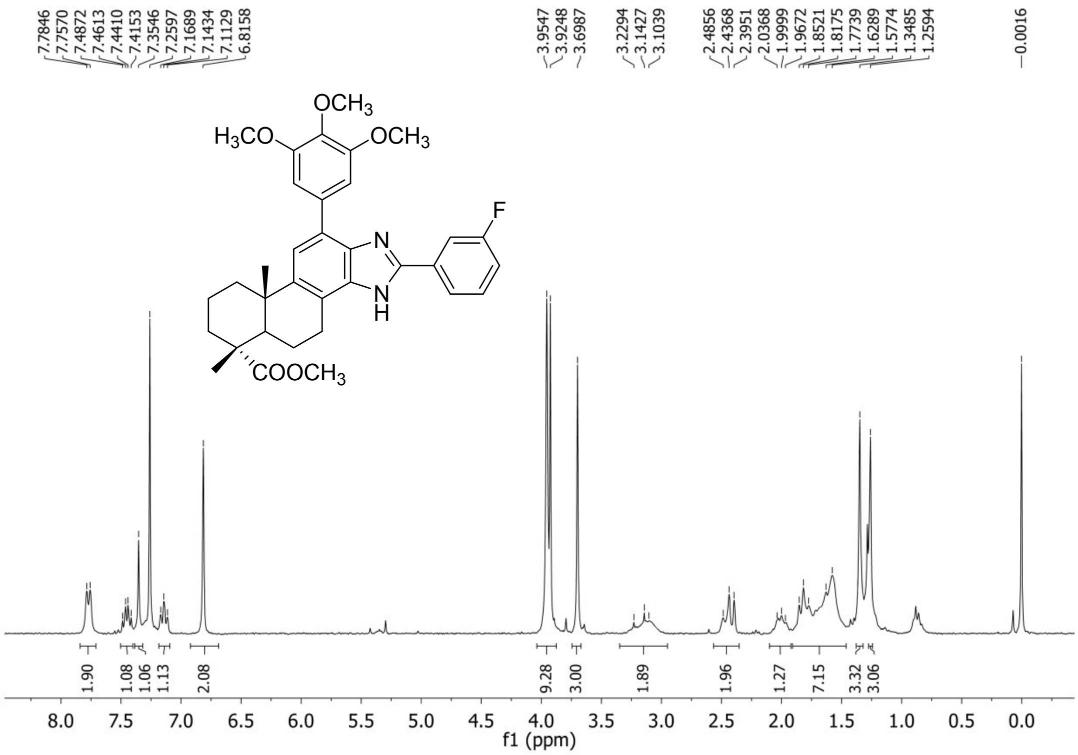

Fig. S29 ^{1}H NMR spectrum of compound **7d** (300 MHz, $CDCl_3$)

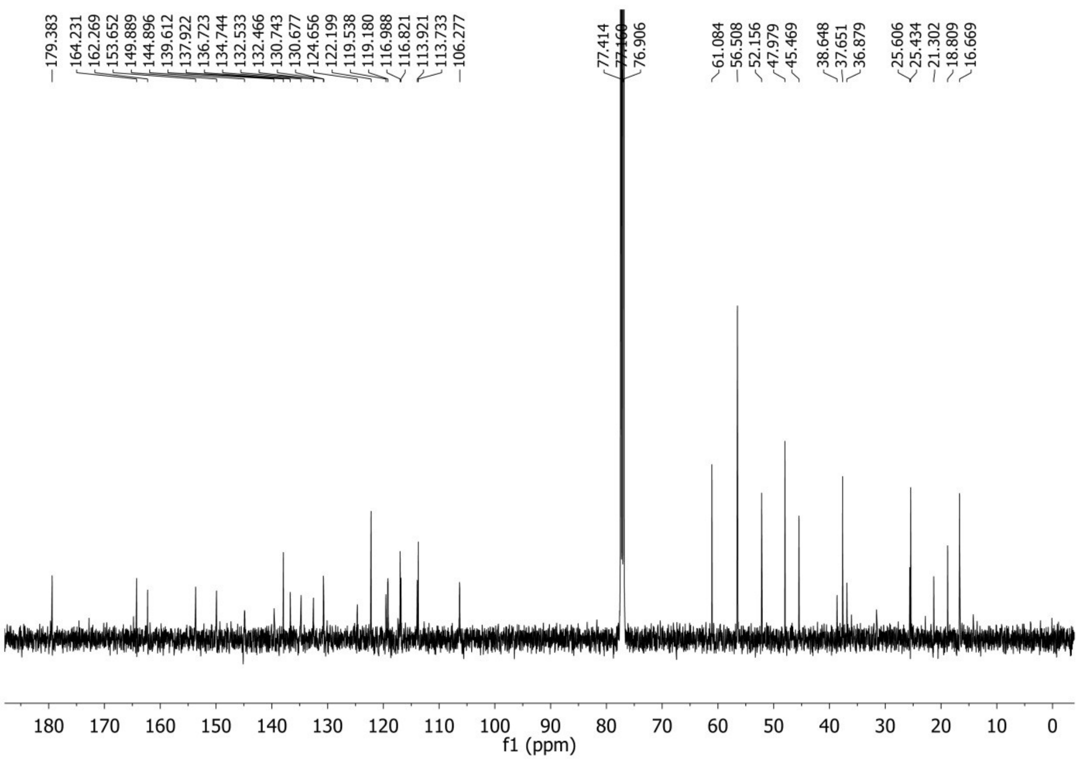

Fig. S30 ^{13}C NMR spectrum of compound **7d** (125 MHz, $CDCl_3$)

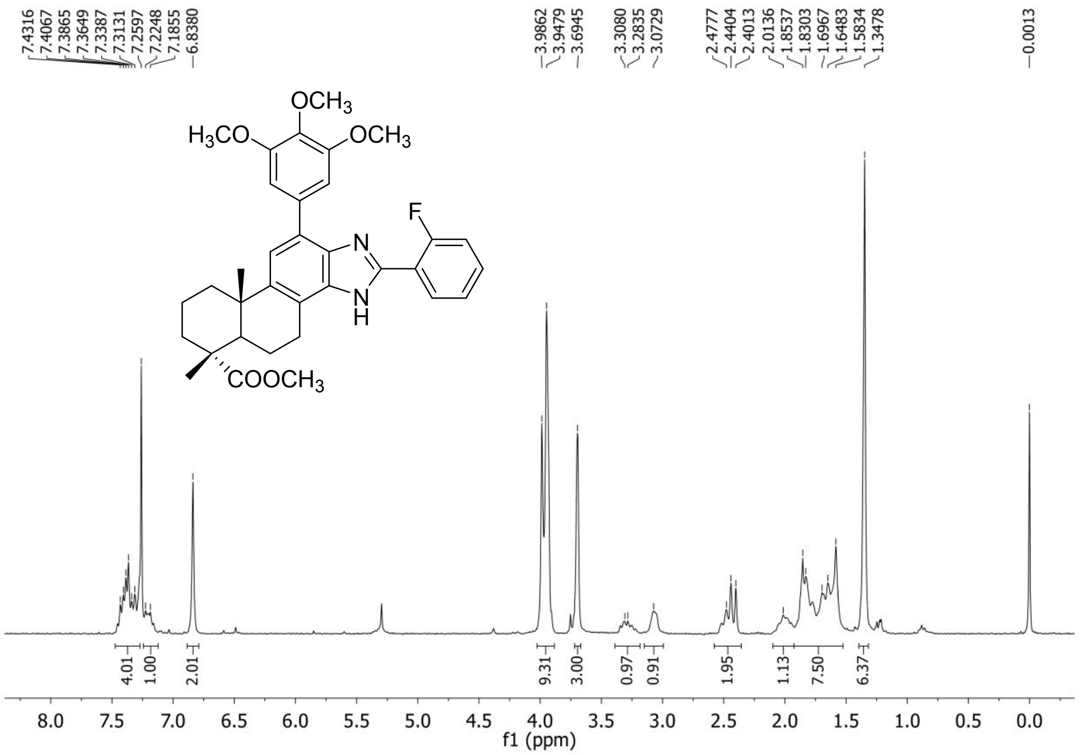

Fig. S31 ^{1}H NMR spectrum of compound **7e** (500 MHz, $CDCl_3$)

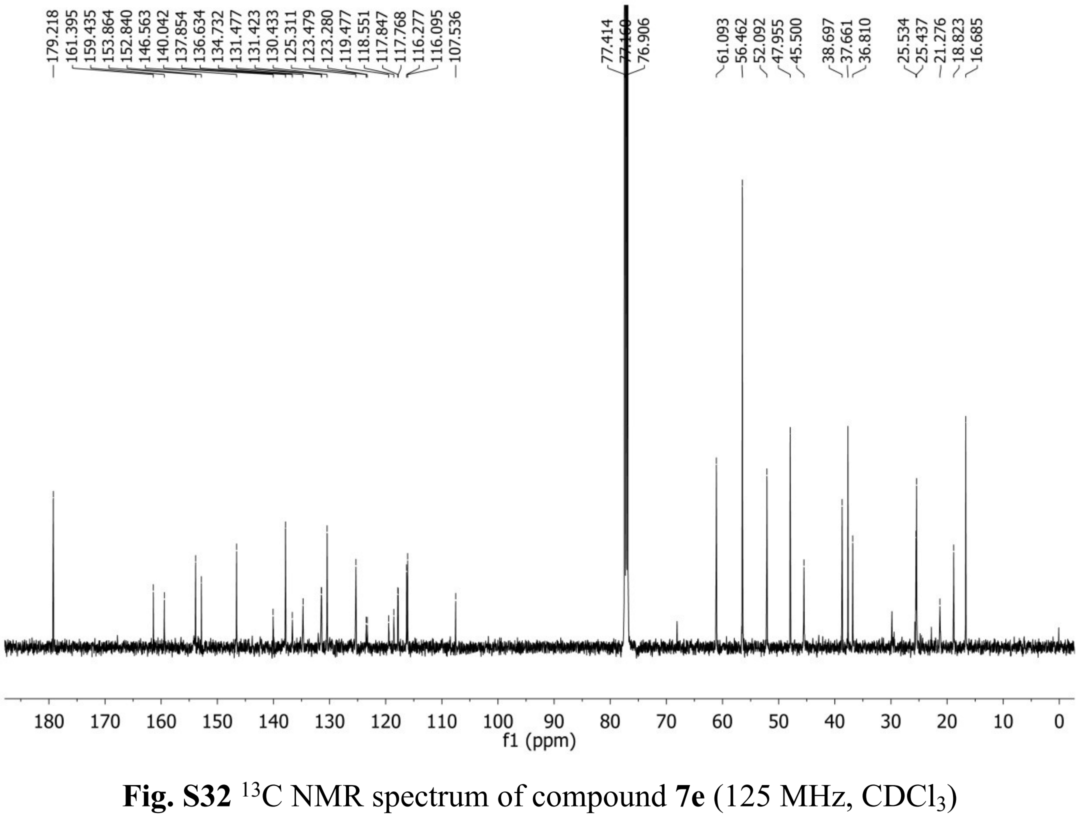

Fig. S32 ^{13}C NMR spectrum of compound **7e** (125 MHz, $CDCl_3$)

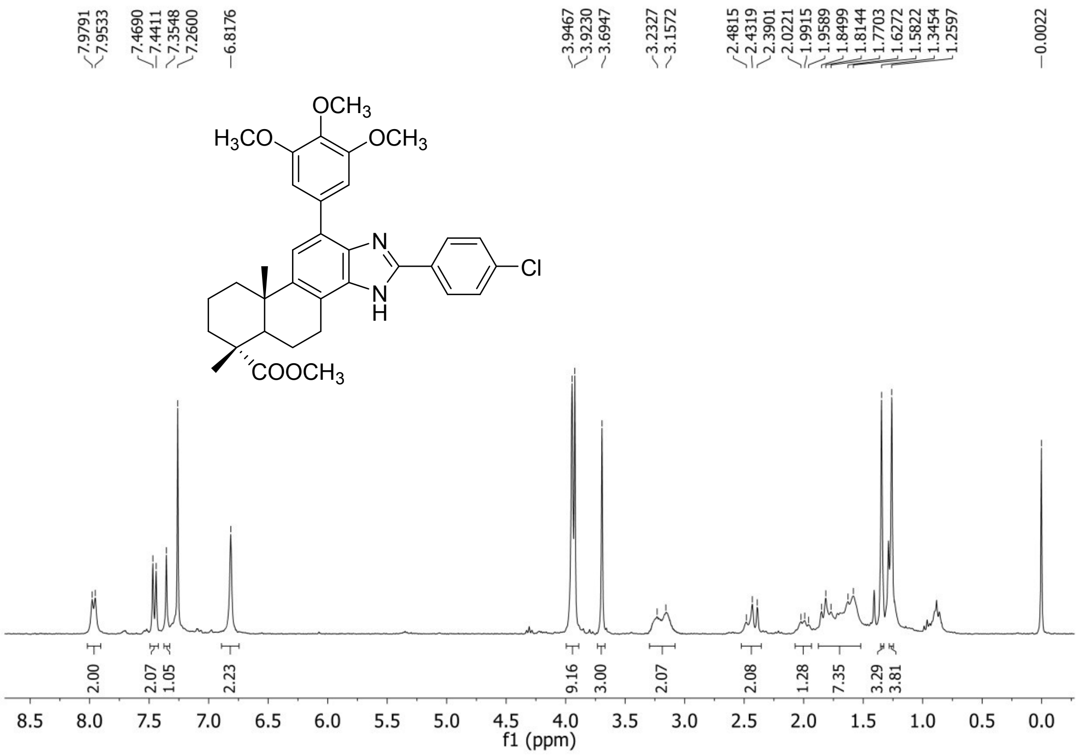

Fig. S33 ^{1}H NMR spectrum of compound **7f** (500 MHz, $CDCl_3$)

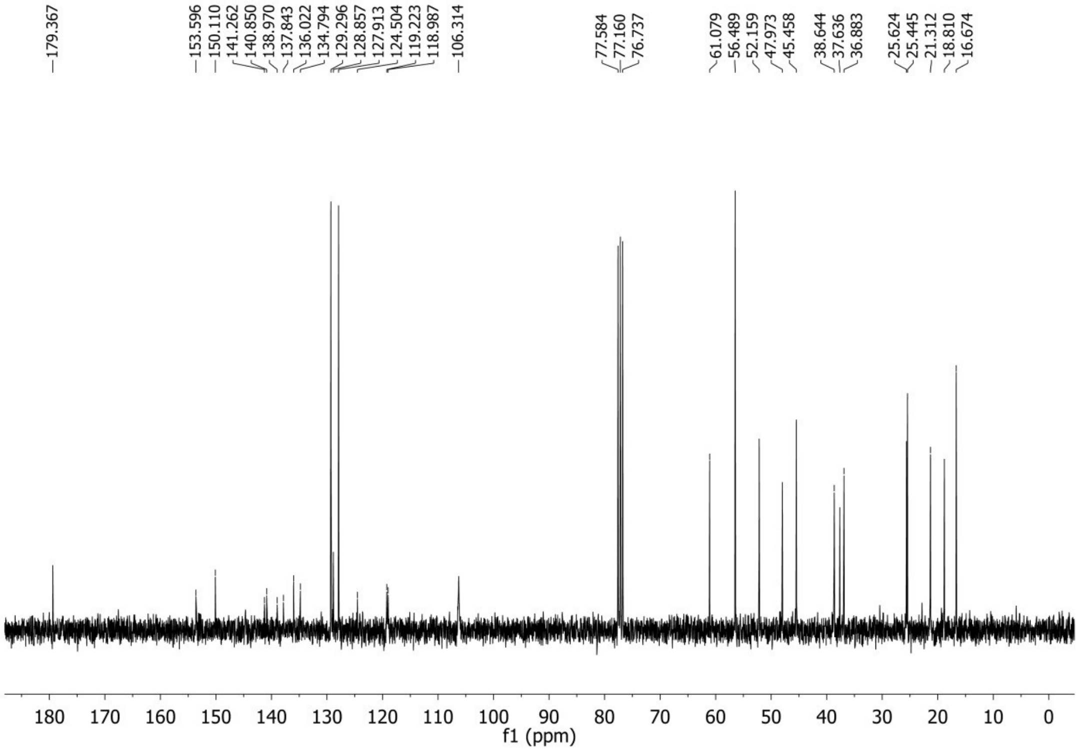

Fig. S34 ^{13}C NMR spectrum of compound **7f** (75 MHz, $CDCl_3$)

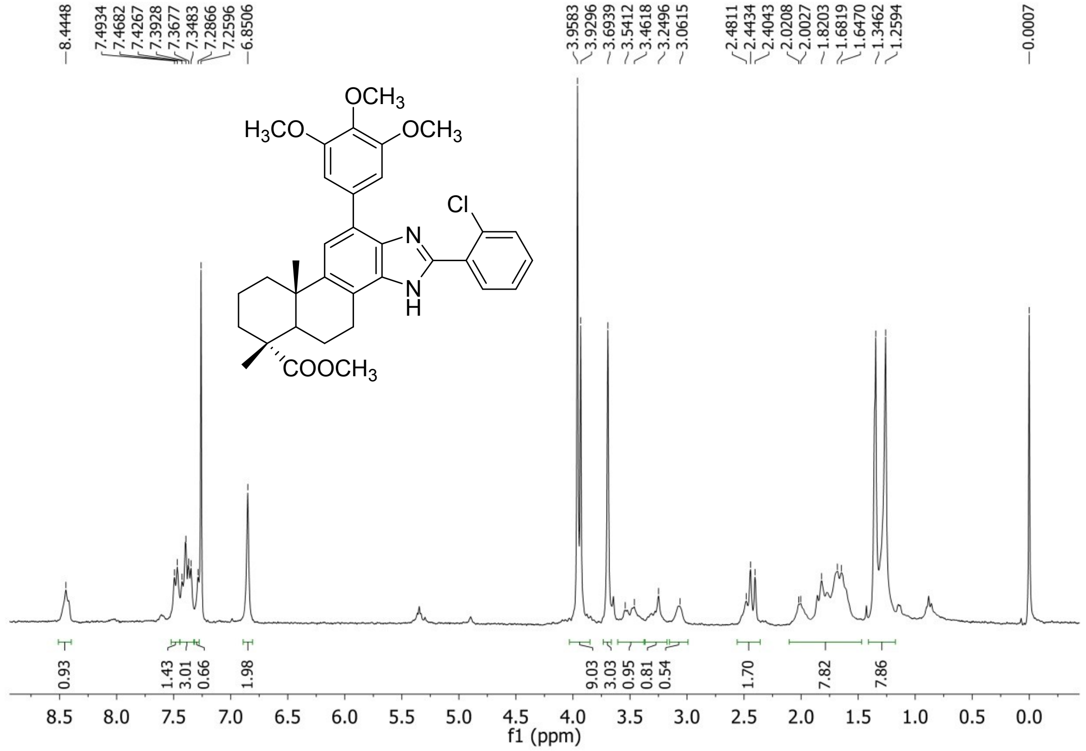

Fig. S35 ^{1}H NMR spectrum of compound **7g** (500 MHz, $CDCl_3$)

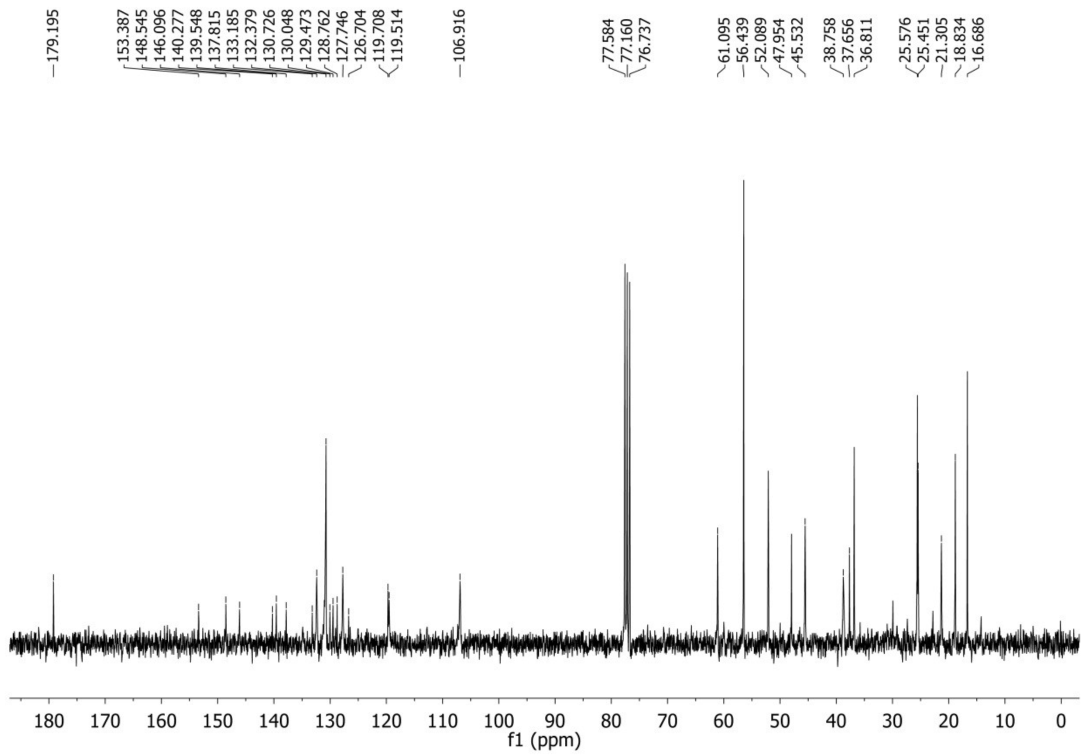

Fig. S36 ^{13}C NMR spectrum of compound **7g** (75 MHz, $CDCl_3$)

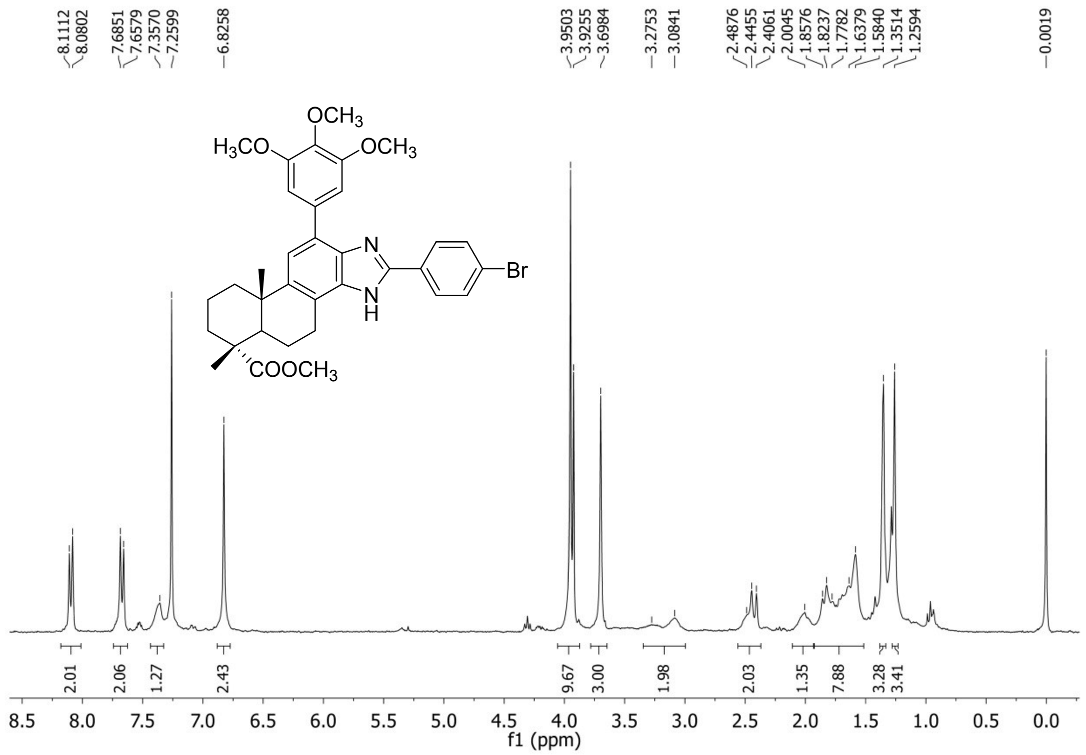

Fig. S37 ^{1}H NMR spectrum of compound **7h** (300 MHz, $CDCl_3$)

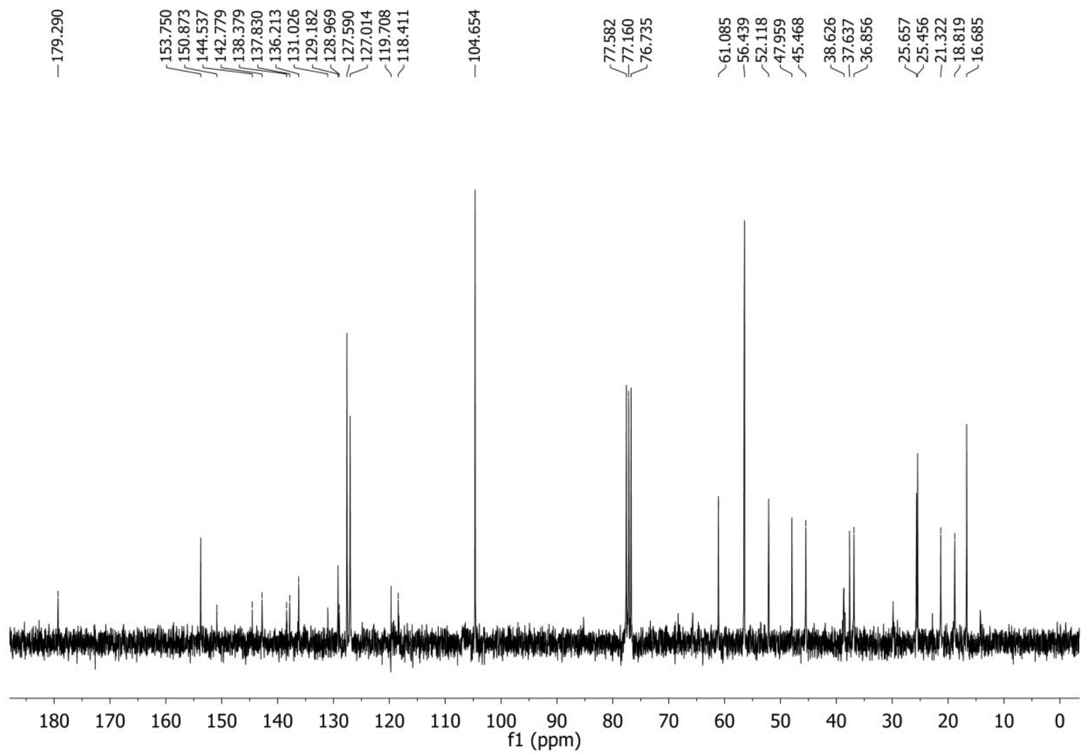

Fig. S38 ^{13}C NMR spectrum of compound **7h** (75 MHz, $CDCl_3$)

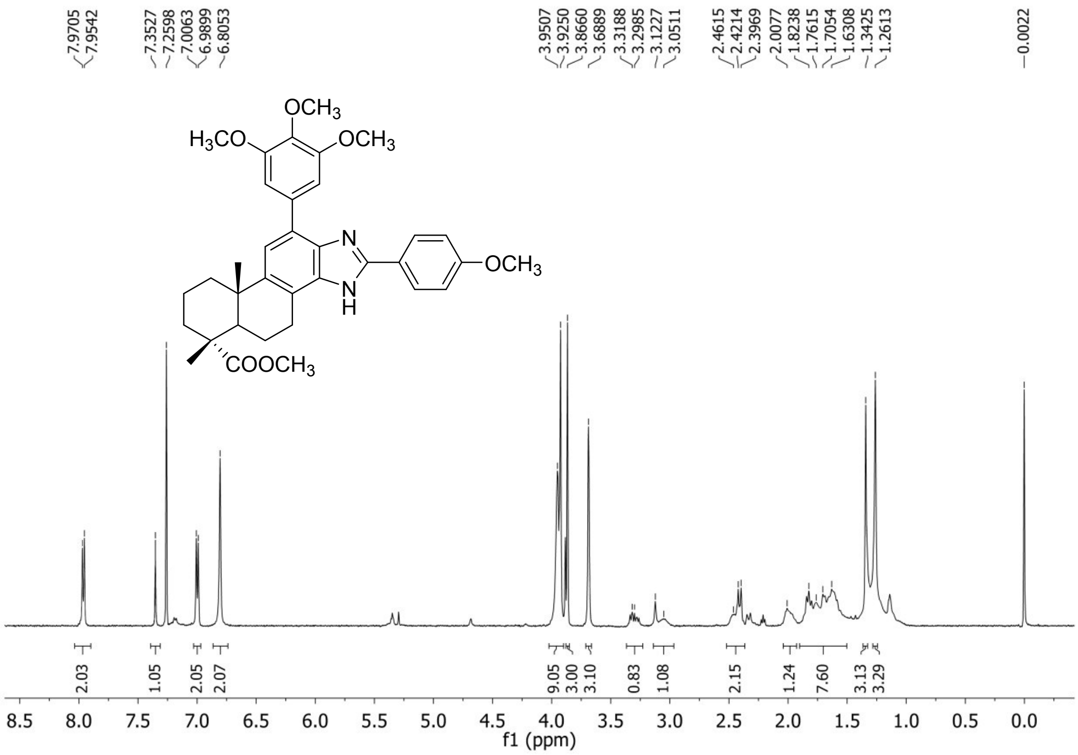

Fig. S39 ^{1}H NMR spectrum of compound **7i** (500 MHz, $CDCl_3$)

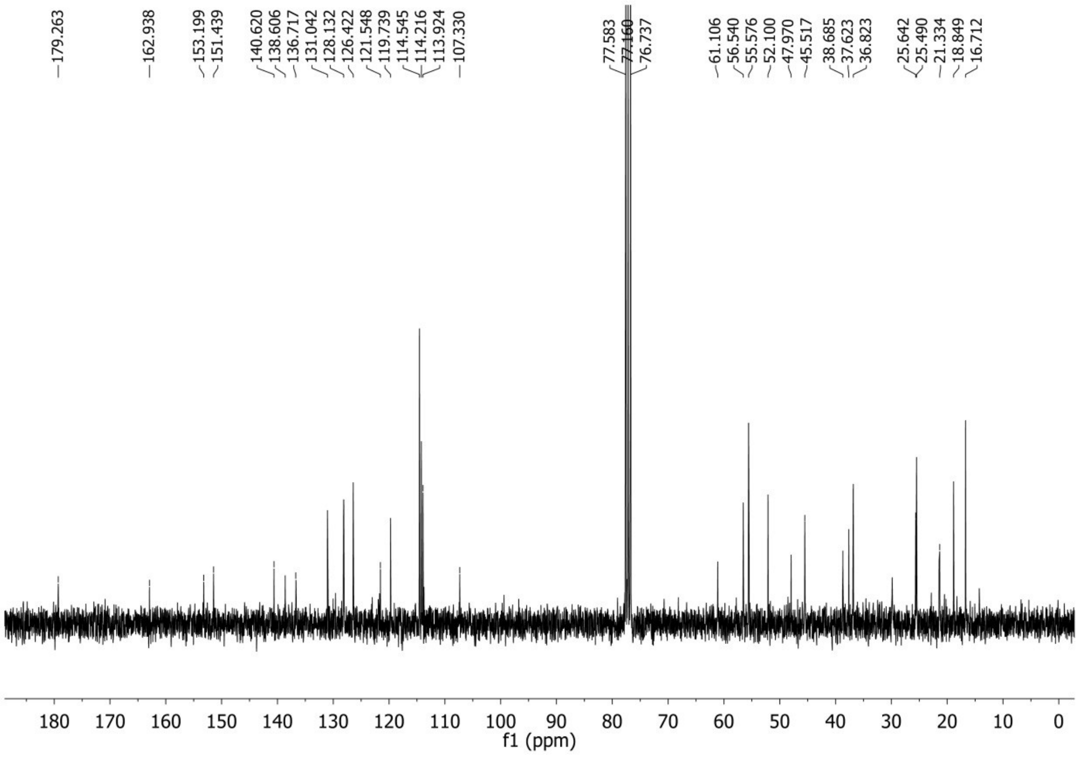

Fig. S40 ^{13}C NMR spectrum of compound **7i** (75 MHz, $CDCl_3$)

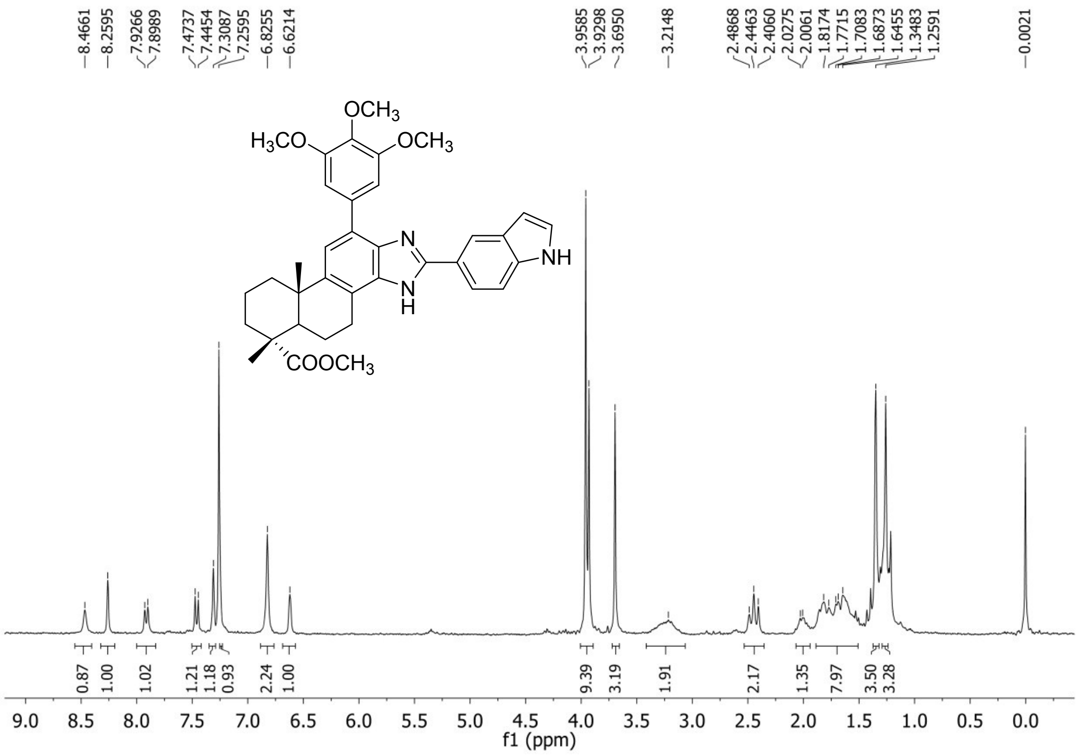

Fig. S41 ^{1}H NMR spectrum of compound **7j** (500 MHz, $CDCl_3$)

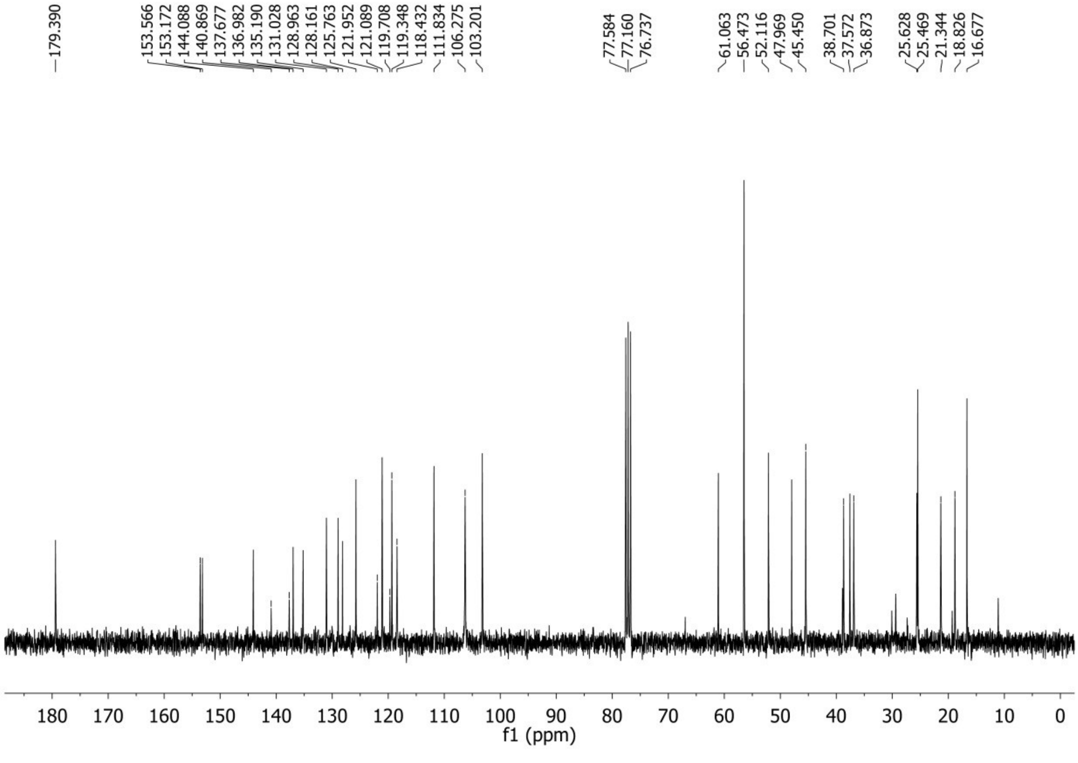

Fig. S42 ^{13}C NMR spectrum of compound **7j** (75 MHz, $CDCl_3$)

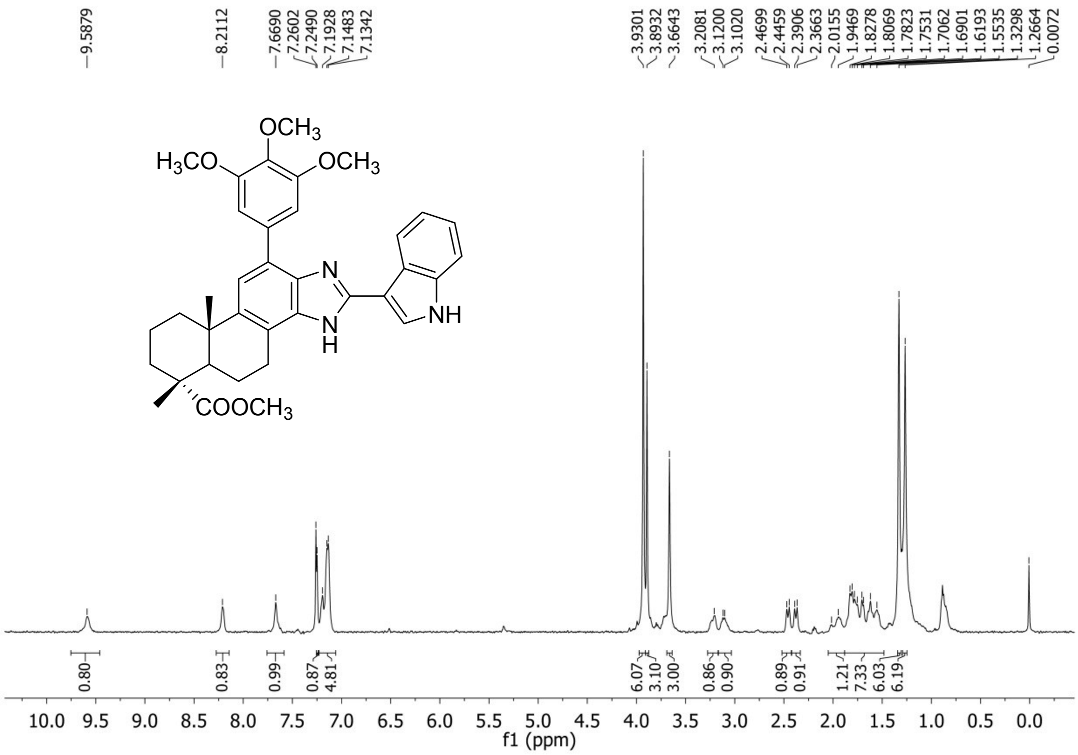

Fig. S43 ^{1}H NMR spectrum of compound **7k** (500 MHz, $CDCl_3$)

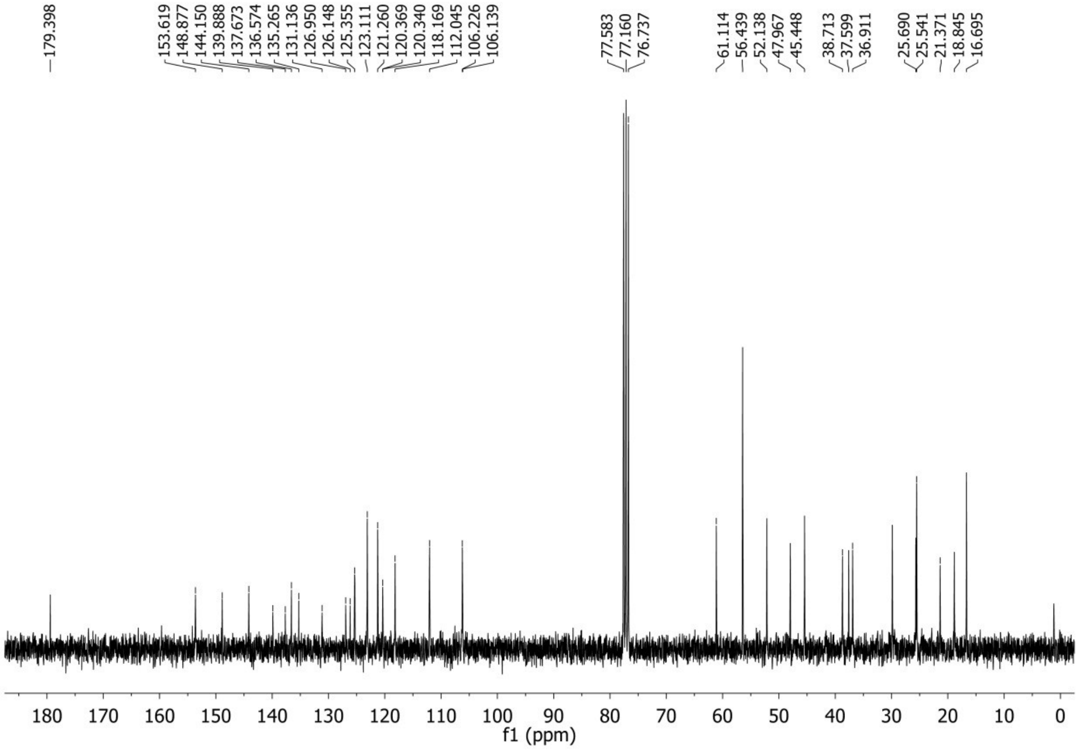

Fig. S44 ^{13}C NMR spectrum of compound **7k** (75 MHz, $CDCl_3$)
